# Benthic diatoms and phytoplankton diversity in urban streams and ponds: charting a course for conservation

**DOI:** 10.1007/s10531-026-03305-z

**Published:** 2026-04-17

**Authors:** A. T. Castro-Castellon, M. J. Hill, E. Conroy, A. Daw, H. Gurung, J. S. Hernández-Avilés, S. J. Hobbs, V. H. Salinas-Camarillo, I. Thornhill

**Affiliations:** 1 School of Geographical Sciences, University of Bristol, University Road, Bristol, BS9 1SS UK; 2 Department of Agriculture and Environment, Harper Adams University, Newport, TF10 8NB UK; 3 School of Sciences, Bath Spa University, Newton St. Loe, Bath, BA2 9BN UK; 4 Laboratory of Limnology, FES Zaragoza, Universidad Nacional Autónoma de México, Batalla 5 de Mayo S/N, Ejército de Oriente, Iztapalapa, Mexico City, 09230 Mexico; 5 School of Environment, Education and Development (SEED), University of Manchester, Oxford Road, M13 9PL Manchester, UK

**Keywords:** Freshwater microalgae, Lentic habitats, Freshwater management, Microalgae assemblages, Biodiversity

## Abstract

Benthic diatom and phytoplankton communities are key indicators of freshwater ecosystem health and change but remain understudied. A better understanding of their distribution and abundance drivers is crucial for effective biodiversity assessment and conservation. This study adopts a novel, multi-ecosystem approach to examine the seasonal (2019–2020) diversity of benthic diatom assemblages in urban streams and phytoplankton assemblages in ponds across Bristol (UK) and the local environmental factors shaping these communities. Among ponds, winter recorded significantly fewer phytoplankton taxa than summer and autumn. Among streams, patterns of benthic diatom beta diversity indicated higher turnover in autumn than in winter, and among pond habitats, turnover dominated in the summer and autumn seasons, but nestedness explained more of the composition variation in winter. No significant differences in Local Contributions to Beta Diversity (LCBD) were recorded between seasons for either streams or ponds. However, among streams, LCBD was consistently high at sites with elevated phosphorus, indicating how nutrient enrichment can shape unique assemblages. In streams, nitrate emerged as the principal driver of diatom diversity and composition during the autumn season, whilst, pH, ammonium, zinc, and potassium distinguished diatom communities during the winter. In ponds, pH influenced phytoplankton assemblages in summer, possibly through nutrient and inorganic carbon availability changes. However, no strong environmental–species relationships were identified in autumn or winter, suggesting biotic interactions may be more influential during these seasons. We highlight the need for more comprehensive, long-term monitoring for the inclusion of diatoms and phytoplankton in urban freshwater management and conservation strategies.

**Supplementary Information:**

The online version contains supplementary material available at 10.1007/s10531-026-03305-z.

## Introduction

Despite the widely recognised biodiversity and ecosystem service value of freshwaters, their fauna and flora face considerable pressures, primarily from urbanization, climate change and agricultural intensification (Kelly et al. 2009; Reid et al. 2019). Within urban landscapes, freshwater habitats (rivers, streams, ditches, lakes and ponds) are subjected to fragmentation and modification of physical habitat, drastic alterations in water quality and quantity due to water abstraction, diversion, industrial and domestic effluents discharge, and urban runoff, in addition to species invasions (Paul and Meyer 2001; Davies et al., 2008). The cumulative effects of these anthropogenic stressors often result in high concentrations of nutrients, metals, pesticides, pharmaceuticals, and microplastics (Paul and Meyer 2001; Castrignanò et al., 2020; Castro-Castellon et al., 2022), which disrupt natural ecological processes, threatening aquatic life.

Algae represent a highly diverse group of non-monophyletic species, with recent estimates identifying 50,589 living algal species (Guiry and Guiry, 2024), including 16,427 taxonomically valid diatom species constituting nearly one-third of this diversity (Mann and Vanormelingen 2013; Mann, 1999). Green algae (Chlorophyta) and blue-green algae (Cyanobacteria) are also significant groups, comprising 6,851 and 5,723 living species respectively (Guiry and Guiry, 2024). Freshwater ecosystems depend on primary producers, with algae at the base of the food web, providing vital energy sources and contributing up to 80% of the energy input in streams (Walsh et al. 2001). Benthic diatoms, as a dominant component of algae, contribute to the global carbon and silicon cycles, accounting for about 20% of global primary production (Field et al., 1998). Phytoplankton, a subset of wandering algae, is responsible for approximately 50% of global oxygen production (Field et al., 1998). Their diversity drives the ecosystem’s capacity for efficient resource use (Ptacnik et al., 2008; Abonyi et al. 2018).

Freshwater benthic diatoms and phytoplankton are two of the five biotic parameters (including macrophytes, macroinvertebrates and fish) considered by the European Water Framework Directive (WFD) to assess ecological status and detect environmental pressures (Kelly and Whitton 1995; Kelly et al. 2009; Kahlert et al. 2012). Benthic diatoms and phytoplankton have a wide range of use as bioindicators because they are present in all rivers, streams, lakes and ponds across a wide gradient of hydrological or geomorphological characteristics. The composition of benthic diatom communities is influenced by changes in water quality, including variations in nutrient concentration, acidification, biological oxygen demand, salinity, alkalinity, and conductivity. This sensitivity allows them to be utilized in developing various indices for assessing water quality. Some commonly used indices include the Specific Polluosensitivity Index (IPS, Cemagref, 1982), the Trophic Diatom Index (TDI, Kelly and Whitton 1995; Kelly et al. 2009), and the Biological Diatom Index (IBD, Prygiel and Coste, 1999).

Phytoplankton communities have specific preferences for certain ecological conditions and respond quickly to disturbances making them important indicators of environmental change (Salmaso et al., 2014). Moreover, phytoplankton communities are subject to distinct seasonal and interannual changes in response to climate change, facilitating the identification of community shifting with the subsequent disruption of ecosystem processes and environmental services (Winder and Cloern 2010). However, most research on phytoplankton communities has predominantly centred on the role of nutrient availability—particularly phosphorus—as a key driver of their productivity (Ptacnik et al., 2010). The proliferation of certain cyanobacteria species is closely linked to eutrophic conditions, where excess nutrients promote their dominance (Downing et al. 2001; Jeppesen et al. 2005) and to warmer water temperatures potentially enhancing their growth and competitive advantage over other phytoplankton groups (Paerl and Huisman 2008; Winder and Sommer 2012). Ecological responses from long-term studies have documented significant changes in phytoplankton communities in the United Kingdom, attributed to climate change (1960–2012) (Thackeray et al. 2016) and human activities (Maberly et al. 2020a, b). Despite their ecological significance and role as indicators of ecosystem changes, studies on benthic diatom assemblages and phytoplankton communities in freshwater environments remain limited, particularly in urban landscapes. Further understanding of the factors that affect their abundance and distribution is needed to identify appropriate conservation measures and to increase their effectiveness as ecosystem health indicators (González-Paz et al., 2020).

Most urban research on freshwater biodiversity has focused on fish (Tóth et al. 2019), amphibians (Sauer et al., 2022) and macroinvertebrates (Thornhill et al., 2017; Heino et al. 2015). The literature in urban streams for both microalgae communities focuses on alpha diversity, reporting data on diversity, evenness and species richness (Jüttner et al., 2012; Teittinen et al. 2015; Tornés et al., 2018; González-Paz et al., 2020). Jüttner et al. (2012) studied benthic diatoms from the urban Ely and Taff rivers and found the dominant taxa to be widely distributed taxa, such as *Navicula gregaria*, which is generally found to indicate moderate or poor water quality status in the UK. Telford et al. (2006) analysed the spatial structure of benthic lake diatom assemblages and demonstrated that species richness at both local and regional scales is shaped not only by environmental factors (e.g. water chemistry) but also exhibits substantial variation across geographic distances. A limited number of studies have considered the compositional variation among phytoplankton and diatoms. Ptanick et al. (2010) studied phytoplankton communities in 4,800 urban and rural lakes across Finland, Norway, and Sweden, determining that the dynamics of high beta diversity were driven by strong selection and limited dispersal. Maloufi et al. (2016) studied phytoplankton communities across 50 freshwater bodies in Paris, highlighting that the heterogeneous anthropogenic pressures and connectivity to the hydrological network promoted beta-diversity.

Preserving benthic diatom assemblages, phytoplankton communities, and their functions in aquatic ecosystems requires a thorough understanding of their diversity in both lotic and lentic systems, especially in urban streams and ponds (Hill et al., 2021; Heino et al., 2017). While research on the biodiversity of urban freshwaters is growing, it remains limited relative to other landscapes (Oertli and Parris 2019). Expanding studies on phytoplankton communities and benthic diatoms assemblages within urban freshwaters are needed to uncover these systems’ ecological significance and implement targeted conservation strategies. By resolving the unique biodiversity contributions of small freshwater systems, these efforts can help mitigate biodiversity loss and enhance the resilience of freshwater ecosystems in the face of urbanisation.

This multi-ecosystem study investigates stream benthic diatom and pond phytoplankton across an urban landscape (Bristol, UK). We aim to examine the diversity (alpha, beta and gamma) of benthic diatom and phytoplankton communities from urban streams and ponds, and to quantify the environmental drivers of the local diatom (stream) and phytoplankton (pond) communities to ascertain the conservation value of these urban inland waters. These analyses address the following research questions: (i) What patterns emerge in benthic diatoms (stream systems) and phytoplankton (pond systems), species richness, and composition? (ii) What environmental factors shape benthic diatom composition in stream systems and phytoplankton communities in pond systems?

## Methods

### Study area

This study was conducted in the city of Bristol, UK, where freshwater systems cover 3% of land coverage (streams and ponds), but impermeable surfaces such as roads, buildings and pathways comprise most of the land cover (66%), with green spaces such as parks and gardens contributing 31% of coverage (Bristol Green Capital Partnership 2015). The River Avon is the principal river flowing through Bristol to the west before entering the Severn Estuary. Between 2019 and 2020, the annual air temperature ranged from − 4.6 °C minima (January 2020) to 38.6 °C (July 2019) maxima, with an average of 12.1 °C. Annual precipitation averaged 852.2 mm, with the driest period being April 2020 (20.5 mm) and the wettest February 2020 (120.2 mm) (www.meteoblue.com/en/user/order/historyplus). Sites were selected to capture a socio-economic gradient according to the Index of Multiple Deprivation as part of the RESPiRES (respires.org) project (see Thornhill et al. 2022), thus they also capture a gradient of urbanisation (McDonnell and Hahs 2008).

**Fig. 1:**
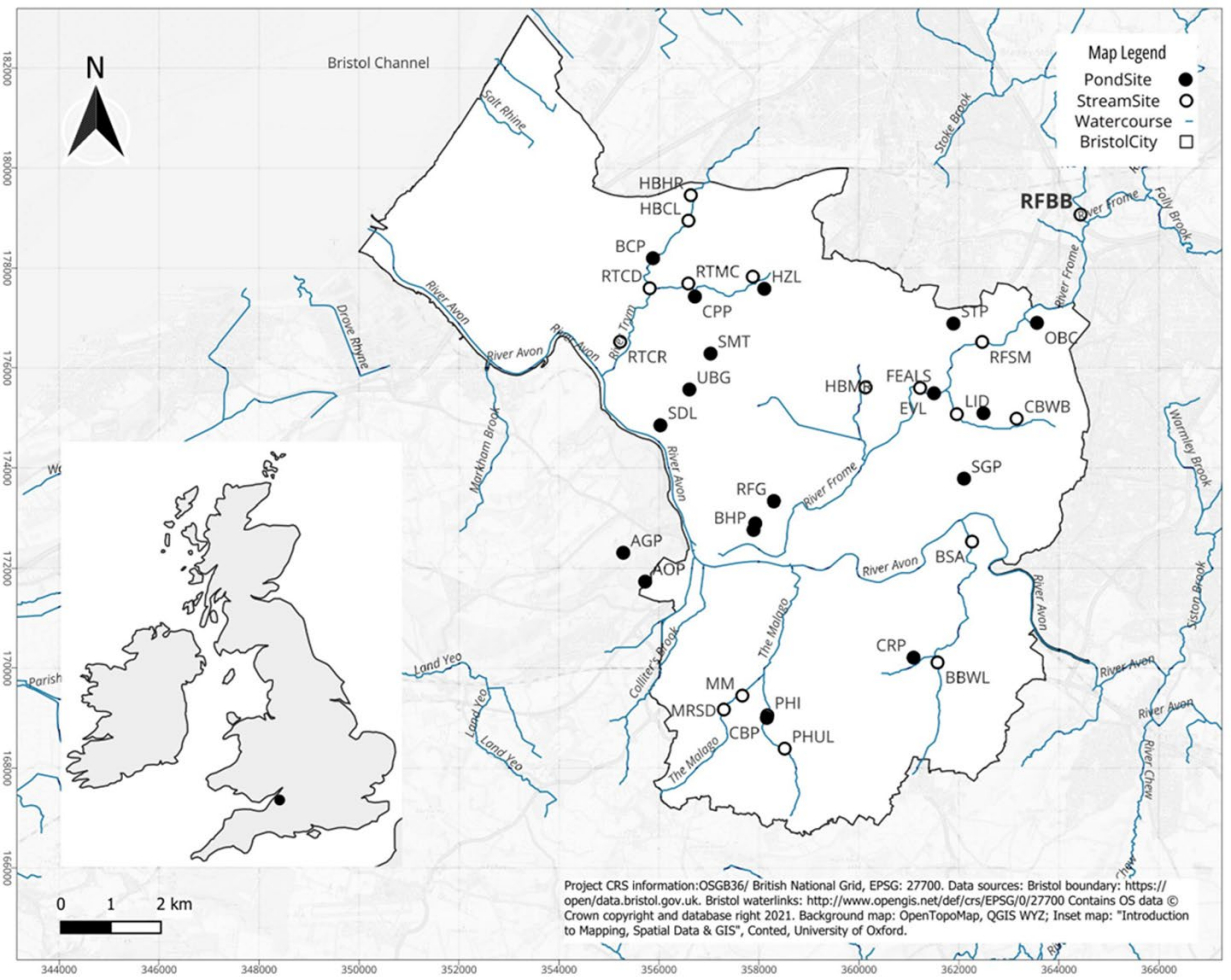
Map of the sites of the 36 surveyed urban ponds (18) and stream (18) sites across Bristol, UK (for full names see Supplementary Information Table S1)

Figure 1 shows the urban stream and pond sites sampled in Bristol. Urban stream sites (*n* = 18; Supplementary Information Table S1) were sampled within the River Avon Urban Operational Catchment (see Environmental Agency 2025 for full classification) and including sections of the River Frome, River Trym, Brislington Brook, The Malago, and other small streams and tributaries. The sampled sites had a mean wetted width of 3.87 m, and depth of 0.26 m. These flowing systems are classified as heavily modified water bodies, having been modified for impoundment, land drainage, flood alleviation engineering, and disconnected from the floodplain along most of their reach. The pond sites sampled (*n* = 18; Supplementary Information Table S1) were distributed across the city, often associated with public parks and privately owned amenity and sites surrounded by semi-improved grassland in high-density built-up areas (residential and industrial). The ponds were all permanent in nature, having a mean area of 2999.9 m^2^ and a mean depth of 0.35 m (Hill et al., 2022). The ponds were all man-made, comprising those installed for conservation (5), ornamental features (4), fishing or boating lakes (4), an ornamental pond (3), a drainage pond for the purpose of watering livestock and an ex-gravel pit.

### Environmental data collection

Sampling occurred across three seasons in 2019/2020: summer (July), autumn (October) and winter (January). At each urban pond and stream sample site, the presence of invasive non-native species (INNS), vegetation structure (absent, < 25%, 25–50%, 50–75%, > 75%) within the riparian zone (extending 15 m landward, 7.5 m up- and down-stream, adapted from the Lake Habitat Survey); the presence of 12 vegetation types (Rowan et al. 2006), water colour, and visual pollution (litter, oily sheen, foam, adapted from the FreshWater Watch methodology (Bishop et al. 2025; Thornhill et al. 2018), were measured from the littoral zone using a rapid assessment tool (Thornhill et al. 2022), (see Supplementary Information Table S2). Some variables were averaged and fixed across seasons: shading (due to some missing observations), land-use within 500 m (agriculture, urban, woodland) according to Land Cover Map 2020 (Morton et al. 2021), fish presence or absence, or average observed visual pollution (Supplementary Information Table S2).

Water quality was assessed at stream and pond site within each season. Dissolved oxygen (%), pH, temperature (^o^C) and conductivity (µScmˉ¹) were recorded using a handheld HACH multiparameter sensor. At each site triplicate water samples were collected to quantify water chemistry including ammonium NH_4_-N (mg Lˉ¹), nitrate NO₃-N (mg Lˉ¹), orthophosphate PO_4_-P (µg Lˉ¹), total phosphorus TP (µg Lˉ¹), alkalinity (mg Lˉ¹ CaCO3) and total suspended solids (mg Lˉ¹ TSS) as described in Hill et al. (2022). A composite sample of 100 ml from each site was acidified with 1 ml HNO_3_ 2% and sent for metal analysis (Al, Fe, Mg, Mn, Pb, Zn, K and Na) to a UKAS Laboratory (United Kingdom Accreditation Service). Three water depth (m) measurements were taken at each site for 1 min (streams: equidistantly across a stream cross section, ponds: within the sampled littoral area), and the mean was derived. From the Rapid Assessment Tool, we used data from the coverage of each site by woody debris (%), emergent, submerged, or floating macrophytes (%), and open water (%) was also estimated. Water velocity (m sˉ¹) (Geopacks Advanced Stream Flow Meter, Devon, UK) was recorded only from urban stream sites.

### Benthic diatom and phytoplankton data collection

Each stream site was sampled for diatoms in autumn (2019) and winter (2019–2020). No diatom samples were collected during the summer, which is why summer environmental data from the streams was not included in the subsequent statistical analysis. Benthic diatom sampling adhered to European standards CEN 13946:2003 and CEN 14407:2004 (European Committee for Standardization, 2003; 2004). At least five cobbles were randomly selected from the water, and a defined area of approximately 25 cm^2^ was gently rinsed with water and brushed off with a toothbrush from each. Samples were immediately preserved with 1% Lugol. In the laboratory, samples were oxidised first using hydrogen peroxide and second using sulphuric acid and potassium dichromate. Samples were thoroughly rinsed with distilled water between treatments. Permanent slides were then mounted using Naphrax^®^ (refractive index = 1.74, Brunel Microscopes Ltd., Chippenham, Wiltshire, UK). Diatom analysis employed a light microscope (Nikon Eclipse 80i, Tokyo, Japan) with a micrometric scale at ×1000 magnification, enumerating 300–400 valves per slide. While 36 samples were initially collected, one site was removed as no diatom material was observed on the corresponding slide. Diatom species were identified to genus or species level according to Blanco et al. (2007), Bellinger and Sigee (2015), Lange-Bertalot et al. (2017) and Spaulding et al. (2021). Nomenclature and some taxonomic concepts were updated using AlgaeBase (Guiry and Guiry, 2024).

There are challenges in distinguishing certain species, such as *Platessa conspicua* (Ant. Mayer) Lange-Bertalot 2017, and similar diatoms like *Platessa oblongella* (Østrup) Wetzel et al. 2017; and *Ulnaria acus* Kutzing 1844 (including its synonym *Fragilaria ulna* var. *acus* Lange-Bertalot 2017). Despite our efforts to align findings with existing ecological descriptions, the difficulties in identifying these species raise concerns about misidentification. For instance, *Synedra acus* is known as a planktonic species that tolerates varying pH and nutrient levels. A deeper understanding of the ecological requirements and distribution of *P. conspicua* and related species is necessary.

Each pond site was sampled for phytoplankton in the summer and autumn (2019), and winter (2019–2020). All samples were preserved in Lugol 1% and a composite sample was prepared mixing all the subsamples and subsampling 100 ml and maintained refrigerated at 4–5 °C. Phytoplankton quantification was conducted using a Sedgwick-Rafter counting chamber (1 mL volume) and light microscopy (x200 magnification) following Bellinger and Sigee (2015). Counting followed a systematic pattern (top-to-bottom, left-to-right) across a minimum of 30 randomly selected grid squares. Cells within each square, as well as those contacting the right and bottom borders, were enumerated. Sparse samples aimed for 300–400 total cells across the entire chamber, while dense samples were diluted 1:10. Colonies and filaments from Cyanobacteria and Chlorophyta were obtained but represented as single units in final calculations to reduce potential counting error (Brierley et al., 2007). Cells lacking internal structures (indicative of death) were excluded. Phytoplankton identification was made to genus level according to John et al. (2011), and the companion website Carter et al. (2016), nomenclature and taxonomic concepts were updated using AlgaeBase (Guiry and Guiry 2024).

### Statistical analysis

Kruskal-Wallis tests were used to test if there were statistically significant differences between seasons in the urban pond (summer, autumn, winter) and stream (autumn, winter) sites. Where there were significant differences, the Nemenyi post-hoc test (using the *kwALLPairsNemenyiTest* function in the PMCMRPlus package: Pohlert et al. 2024) was used to determine which specific groups differed from each other. Similarly, to examine seasonal differences in taxonomic richness (alpha diversity) among pond (phytoplankton) and stream (diatom) sites Kruskal-Wallis and Post hoc Nemenyi tests were employed.

Seasonal differences in diatom community composition among streams and phytoplankton communities among ponds were statistically examined using a Pairwise Permutational Analysis of Variance (PERMANOVA, Martinez Arbizu *Pers Comm*), and visualised using NMDS (based on the Sorensen dissimilarity metric) using the *metaMDS* function from the R vegan package (Oksanen et al. 2025). To examine the variation in community composition within each season for the urban streams and ponds, homogeneity of multivariate dispersions was undertaken using the *betadisper* function in the vegan package. To compare seasonal differences in multivariate dispersion among urban stream and pond sites an ‘Analysis of Variance’ (ANOVA) was undertaken. Total beta-diversity and the contribution of the turnover and nestedness components were calculated using the *beta.div.comp* function (using the Baselga family Jaccard based indices) in the adespatial package (Dray et al. 2024). The Local Contributions to Beta Diversity (LCBD) for each pond and stream site was then calculated using the *Beta.div* function (using the Hellinger distance) in the adespatial package (Dray et al. 2024). LCBD measures the contribution of each sampling unit (e.g. site) to the overall beta diversity of a dataset (Legendre and De Caceres 2013).

The influence of physical or chemical factors upon phytoplankton and diatom assemblages in ponds and streams respectively was explored using redundancy analysis (RDA). First, all variables were assessed for completeness and for covariance. An independent variable was dropped where any two were correlated by ρ ≥ 0.80 (Spearman’s Rank) within the pooled dataset (i.e., when averaged across all seasons sampled), retaining the most ecologically meaningful variable (see Supplementary Information Table S3). To retain all sites where algae communities were sampled, a small number of NA values were replaced with medians. This was applied to no more than two cases (11% of sites) within any one variable. All variables were standardized prior to RDA analysis to a mean of 0 and a standard deviation of ± 1. All phytoplankton and diatom abundances were square-root transformed to account for outliers. A stepwise selection procedure (forward and backward selection) was employed to select the best model and environmental variables that significantly (*P* < 0.05) explained the variance in phytoplankton and diatom assemblages using the *ordistep* function in the vegan package (Oksanen et al. 2025), which uses permutation-based significance tests (999 permutations). RDAs were performed for each season separately, and with a pooled dataset using average values from all seasons, for both independent and dependent variables. Two sites (BCP and BHP) were removed from the autumn phytoplankton analysis as too few algal cells were sampled. One site (AOP) was removed from the winter analysis for the same reason, as was HBHR removed from the autumn analysis of diatom assemblages.

## Results

### Environmental characteristics among urban streams and pond sites

Concentrations of orthophosphate (PO4-P), nitrate (NO3-N), and metals such as aluminium, iron, and lead within stream sites were significantly higher in autumn compared to winter (Post hoc Nemenyi tests, *p* < 0.05; Tables 1 and 2). During the summer, the sites with the highest orthophosphate concentration were Malago Manor (MM), measuring 0.343 mg Lˉ¹, and Malago Local Natural Reserve (MRSD), measuring 0.333 mg Lˉ¹. In the autumn, the highest orthophosphate concentration, 0.328 mg Lˉ¹ was found at Pigeonhouse Stream (PHI). Ammonium (NH4-N), flow velocity, and total suspended solids (TSS) were also significantly higher in winter than in autumn (*p* < 0.05; Table 2). On the three sampling occasions, a site consistently high for NH4-N was Coombe Brook (CBWB), with 43.33, 68.81, and 124.3 mg Lˉ¹ for summer, autumn, and winter respectively (Table 2). Across the urban streams, there was minimal coverage of submerged, emergent, floating and free-floating macrophytes (Table 1)with only four sites having a small (< 20%) coverage of macrophytes, and as a result, seasonal differences for these were not analysed.

Significant seasonal differences in some environmental variables were recorded among the urban ponds (Table 2). Post hoc Nemenyi tests identified that TP, temperature and ammonium (NH4-N) were significantly higher in summer than in autumn and winter (*p* < 0.05) among the urban pond habitats (Table 2). Nitrate (NO3-N) was significantly lower in autumn than in winter (*p* < 0.05) and was significantly lower in summer than in autumn (*p* < 0.001). Aluminium was significantly higher in autumn than in winter (*p* < 0.001). Iron and lead were significantly lower in winter than in summer and autumn (*P* < 0.05). The percentage coverage of floating macrophytes was significantly higher in summer than in winter (*p* < 0.05), whilst open water was significantly lower in summer compared to autumn and winter (*p* < 0.05;Table 2).

**Table 1:** Data items to be extracted

			**PO4-P mg Lˉ¹**	**NO3-N mg Lˉ¹**	**NH4-N mg Lˉ¹**	**Alkalinity Equ. Lˉ¹**	**pH**	**Cond µS cmˉ¹**	**Temp oC**	**DO %**	**Depth m**	**Flow m sˉ¹**	**TSS mg Lˉ¹**	**Al mg Lˉ¹**	**Fe mg Lˉ¹**	**Pb mg Lˉ¹**	**Mg mg Lˉ¹**	**Mn mg Lˉ¹**	**K mg Lˉ¹**	**Na mg Lˉ¹**	**Zn mg Lˉ¹**	**Submer- ged %**	**Emergent %**	**Floating %**	**Free Floating %**	**Open %**	**Woody Debris %**
**Stream**	**Summer**	Mean	**0.14**	**5.79**	**5.31**	**NA**	**7.81**	**629.8**	**15.65**	**84.6**	**0.17**	**0.23**	**9.23**	**0.05**	**0.063**	**0.001**	**11.02**	**0.02**	**6.92**	**30.50**	**0.045**	**0.06**	**0.81**	**0.63**	**0.00**	**98.50**	**3.25**
		Med.	0.11	2.18	1.76	NA	7.88	573.0	15.40	88.6	0.12	0.10	7.90	0.054	0.054	0.001	8.55	0.01	4.6	27.00	0.044	0.00	0.00	0.00	0.00	100.00	0.00
		SD	0.10	6.30	15.41	NA	0.61	264.4	0.90	13.0	0.12	0.21	4.78	0.022	0.037	0.001	8.17	0.02	7.77	14.28	0.021	0.24	2.43	1.65	0.00	2.85	6.77
		Min.	0.01	0.00	0.69	NA	5.57	269.0	14.23	39.9	0.04	0.01	2.60	0	0	0.000	3.2	0.004	2.4	11.00	0	0.00	0.00	0.00	0.00	90.00	0.00
		Max.	0.34	19.63	68.81	NA	8.42	1443.0	17.73	96.3	0.43	0.76	22.00	0.085	0.15	0.002	37	0.08	38	75.00	0.084	1.00	10.00	5.00	0.00	100.00	25.00
	**Autumn**	Mean	**0.15**	**16.94**	**6.54**	**285.4**	**8.13**	**881.8**	**11.05**	**90.6**	**0.22**	**0.11**	**8.02**	**0.03**	**0.05**	**0.001**	**17.73**	**0.03**	**9.17**	**35.78**	**0.04**	**1.11**	**2.50**	**0.56**	**0.28**	**95.8**	**7.94**
		Med.	0.15	16.70	0.61	260.0	8.14	833.0	11.05	94.3	0.20	0.11	5.37	0.03	0.04	0.001	14.00	0.02	5.65	31.00	0.04	0.00	0.00	0.00	0.00	100.0	5.00
		SD	0.08	6.24	17.37	72.5	0.28	376.2	1.04	10.9	0.17	0.10	6.68	0.02	0.03	0.000	12.01	0.05	11.96	20.87	0.01	4.71	5.49	2.36	1.18	11.4	9.14
		Min.	0.02	4.14	0.50	200.0	7.71	424.0	9.40	59.9	0.02	0.01	1.33	0.00	0.00	0.000	5.30	0.00	1.90	15.00	0.01	0.00	0.00	0.00	0.00	55.0	0.00
		Max.	0.33	28.84	63.37	503.3	8.68	1811.0	12.50	106.1	0.68	0.28	23.31	0.05	0.09	0.002	45.00	0.16	46.00	87.00	0.06	20.00	20.00	10.00	5.00	100.0	30.00
	**Winter**	Mean	**0.08**	**10.97**	**12.33**	**290.0**	**8.36**	**786.5**	**8.05**	**92.7**	**0.27**	**0.35**	**125.7**	**0.01**	**0.01**	**0.000**	**16.13**	**0.04**	**8.59**	**34.78**	**0.03**	**0.29**	**0.59**	**0.00**	**0.00**	**94.1**	**10.50**
		Med.	0.08	5.85	0.88	249.4	8.44	691.5	8.32	93.5	0.26	0.29	27.84	0.01	0.00	0.000	11.00	0.01	4.25	25.50	0.03	0.00	0.00	0.00	0.00	100.0	5.00
		SD	0.05	16.61	33.85	160.9	0.25	314.1	1.83	7.7	0.18	0.29	165.6	0.01	0.02	0.000	12.89	0.08	13.25	22.27	0.01	1.21	2.43	0.00	0.00	24.3	20.82
		Min.	0.01	0.58	0.41	134.7	7.74	486.0	4.87	73.0	0.06	0.01	5.70	0.00	0.00	0.000	4.50	0.01	1.30	11.00	0.02	0.00	0.00	0.00	0.00	0.0	0.00
		Max.	0.20	63.35	124.30	755.30	8.63	1653.0	10.80	105.1	0.74	1.01	457.3	0.03	0.08	0.001	46.00	0.32	50.00	88.00	0.06	5.00	10.00	0.00	0.00	100.0	80.00
**Pond**	**Summer**	Mean	**0.14**	**1.69**	**1.41**	**NA**	**8.37**	**377.1**	**19.37**	**90.3**	**0.43**	**NA**	**52.85**	**0.05**	**0.14**	**0.001**	**9.52**	**0.02**	**4.55**	**19.07**	**0.04**	**26.44**	**22.33**	**15.22**	**12.67**	**44.8**	**6.72**
		Med.	0.08	0.06	1.33	NA	8.15	373.5	19.59	87.7	0.41	NA	29.30	0.05	0.10	0.001	6.05	0.01	4.25	16.50	0.04	10.00	7.50	4.50	0.00	44.0	0.00
		SD	0.24	3.92	0.88	NA	1.01	165.4	2.28	61.1	0.25	NA	89.09	0.03	0.13	0.001	9.02	0.04	4.45	12.23	0.02	32.30	27.41	21.00	31.24	35.2	13.15
		Min.	0.02	0.00	0.26	NA	7.23	116.0	15.63	6.5	0.13	NA	0.70	0.02	0.04	0.000	1.70	0.00	0.31	2.00	0.01	0.00	0.00	0.00	0.00	0.0	0.00
		Max.	1.07	13.49	3.76	NA	10.34	660.0	22.50	203.1	0.97	NA	376.7	0.12	0.44	0.002	36.00	0.15	20.00	45.00	0.09	100.00	90.00	70.00	100.00	95.0	50.00
	**Autumn**	Mean	**0.19**	**5.59**	**0.64**	**167.2**	**8.03**	**445.1**	**10.71**	**75.6**	**0.50**	**NA**	**14.05**	**0.03**	**0.11**	**0.001**	**9.26**	**0.03**	**4.96**	**18.52**	**0.05**	**30.28**	**18.89**	**4.28**	**5.83**	**78.9**	**7.22**
		Med.	0.11	4.53	0.53	157.0	7.97	475.5	10.77	78.3	0.42	NA	7.80	0.03	0.05	0.001	7.25	0.01	3.85	18.50	0.04	7.50	15.00	1.00	0.00	90.0	5.00
		SD	0.24	4.07	0.45	68.5	0.63	172.0	1.37	30.5	0.41	NA	15.18	0.01	0.18	0.000	6.81	0.03	4.27	8.82	0.02	37.83	18.36	5.69	18.81	29.7	8.95
		Min.	0.00	0.43	0.15	74.0	6.92	136.0	8.30	15.8	0.16	NA	0.16	1.67	0.02	0.000	0.00	1.60	0.00	0.42	4.00	0.02	0.00	0.00	0.00	0.0	2.00
		Max.	1.07	16.67	1.82	287.3	9.53	815.0	13.00	120.3	2.00	NA	2.00	61.11	0.06	0.770	0.00	24.00	0.12	20.00	36.00	0.12	100.00	60.00	20.00	80.0	100.00
	**Winter**	Mean	**0.14**	**1.71**	**0.78**	**170.6**	**8.11**	**421.1**	**6.21**	**76.5**	**0.39**	**NA**	**708.8**	**0.02**	**0.07**	**0.000**	**8.68**	**0.03**	**4.49**	**16.70**	**0.04**	**8.13**	**17.81**	**0.97**	**1.00**	**78.2**	**5.00**
		Med.	0.08	0.61	0.70	179.4	8.10	465.0	7.10	84.1	0.31	NA	7.14	0.01	0.03	0.000	6.80	0.01	3.75	18.00	0.03	2.50	10.00	0.00	0.00	87.5	0.00
		SD	0.28	3.14	0.56	79.1	0.41	175.5	2.29	21.5	0.19	NA	2740	0.02	0.13	0.001	7.05	0.05	3.81	9.07	0.02	13.65	22.58	2.71	2.71	24.9	12.04
		Min.	0.01	0.06	0.05	55.3	7.15	121.0	2.30	16.9	0.17	NA	0.00	0.01	0.00	0.000	1.30	0.00	0.52	3.40	0.02	0.00	0.00	0.00	0.00	10.0	0.00
		Max.	1.18	13.27	2.36	341.3	8.72	700.0	9.70	104.5	0.76	NA	11675	0.09	0.44	0.002	28.00	0.22	18.00	41.00	0.11	50.00	80.00	10.00	10.00	100.0	40.00

**Table 2:** Results from the Kruskal Wallis test examine the statistical differences in stream and pond environmental parameters between summer (pond only), autumn and winter seasons

Environmental variable	Stream				Pond	
	Chi^2^	df	*p* value	Chi^2^	Df	*p* value
PO4-P (mg Lˉ¹)	7.064	1	0.008	2.447	2	0.294
TP (mg Lˉ¹)	0.256	1	0.612	32.555	2	< 0.001
NO3-N (mg Lˉ¹)	12.333	1	< 0.001	18.689	2	< 0.001
NH4-N (mg Lˉ¹)	5.194	1	0.023	10.403	2	0.005
Alkalinity	0.993	1	0.319	0.001	1	0.975
pH	6.331	1	0.012	0.6419	2	0.725
Cond (µS cmˉ¹)	0.901	1	0.343	1.571	2	0.456
Temperature (°C)	19.77	1	< 0.001	45.719	2	< 0.001
Dissolved Oxygen (%)	0.064	1	0.8	0.677	2	0.713
Depth (m)	0.602	1	0.438	0.402	2	0.818
Flow velocity (m sˉ¹)	5.943	1	0.015	NA	NA	NA
TSS (mg Lˉ¹)	14.418	1	< 0.001	4.056	2	0.131
Al (mg Lˉ¹)	14.193	1	< 0.001	26.217	2	< 0.001
Fe (mg Lˉ¹)	10.258	1	0.001	15.687	2	< 0.001
Pb (mg Lˉ¹)	18.591	1	< 0.001	15.203	2	< 0.001
Mg (mg Lˉ¹)	0.344	1	0.558	0.274	2	0.872
Mn (mg Lˉ¹)	0.508	1	0.476	3.953	2	0.139
K (mg Lˉ¹)	3.088	1	0.079	0.289	2	0.866
Na (mg Lˉ¹)	0.049	1	0.824	0.527	2	0.768
Zn (mg Lˉ¹)	0.508	1	0.476	3.049	2	0.218
Emergent Macrophytes	NA	NA	NA	0.366	2	0.833
Submerged Macrophytes	NA	NA	NA	3.810	2	0.149
Floating Macrophytes	NA	NA	NA	7.266	2	0.026
Free floating macrophytes	NA	NA	NA	2.131	2	0.345
Open Water	NA	NA	NA	9.948	2	0.007
Woody Debris %	0.067	1	0.95	3.824	2	0.148

### Urban stream benthic diatom and pond phytoplankton alpha and gamma diversity

**Fig. 2:**
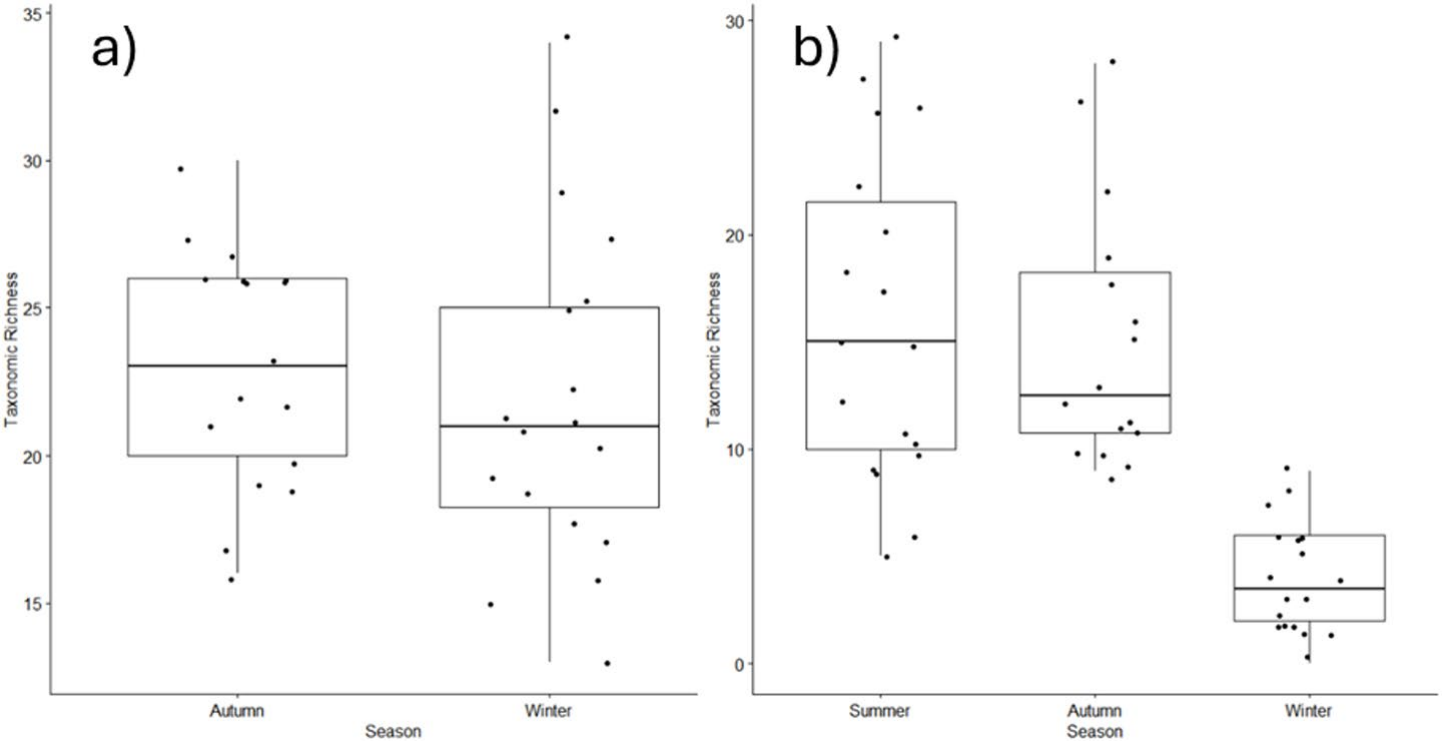
Seasonal richness (boxes show 25th, 50th and 75th percentiles, and whiskers show 5th and 95th percentiles) recorded from (**a**) benthic diatoms in urban streams and (**b**) phytoplankton in ponds

Among flowing sites, 157 benthic diatom taxa (mean = 37.6, median = 36.5, SE = 1.82) were recorded. When all seasons were considered together, the most commonly occurring taxa were *Amphora inariensis* (present within 100% of river sample sites), *Navicula lanceolata* (100%), *Nitzschia palea* (89%), *Cocconeis euglypta* (89%), *Navicula tripunctata* (83%), *Navicula gregaria* (83%) and *Gomphonema olivaceum* (83%). A total of 121 benthic diatom species (mean = 23.1, median = 23, SE = 0.97) were recorded in the autumn, whilst 108 taxa (mean = 21.9, median = 21, SE = 1.36) were recorded from flowing sites in the winter. At an alpha scale, no significant difference in taxonomic richness was recorded between the autumn and winter seasons (Kruskal Wallis, Chi^2^ = 1.124, df = 1, *p* = 0.289; Fig. 2a).

Among pond sites, a total of 77 phytoplankton taxa (mean = 23.6, median = 22, SE = 1.95) were recorded. When all seasons were considered together, the most commonly occurring taxa were Chlorophyte: *Scenedesmus* spp. (present within 100% of urban pond sites), *Pediastrum* spp. (94%) and *Monoraphidium* spp. (78%); Cryptophyte: *Cryptomonas* spp. (94%); planktonic diatoms (Bacillaryophyte) *Nitzschia* spp. (83%) and *Melosira* spp. (72%). A total of 66 phytoplankton taxa (mean = 15.9, median = 15, SE = 1.79) were recorded in the autumn, whilst 62 taxa (mean = 14.2, median = 12, SE = 1.41) were recorded from flowing sites in autumn and 24 phytoplankton taxa were recorded in winter (mean = 3.94, median = 3.5, SE = 0.61). Post hoc Nemenyi test found, at an alpha scale, taxonomic richness was significantly lower in winter compared to the summer and autumn seasons (Kruskal Wallis, Chi^2^ = 31.863, df = 2, *p* < 0.001; Fig. 2b).

### Benthic diatom (stream) and phytoplankton (pond) community composition

When both seasons were considered together, a moderate compositional variation was recorded among urban stream (total beta-diversity: 0.36) and pond (total beta-diversity: 0.35) sites. The total variation in stream and pond composition was mostly explained by species replacement (stream: 80%, pond: 61%), compared to nestedness (stream: 20%, pond: 39%). One stream site (CBWB) recorded a significant LCBD value (0.071, *p* = 0.029), whilst two pond sites recorded significant LCBD values (BCP, 0.075, *P* = 0.01: CPP, 0.074, *p* = 0.01).

**Fig. 3:**
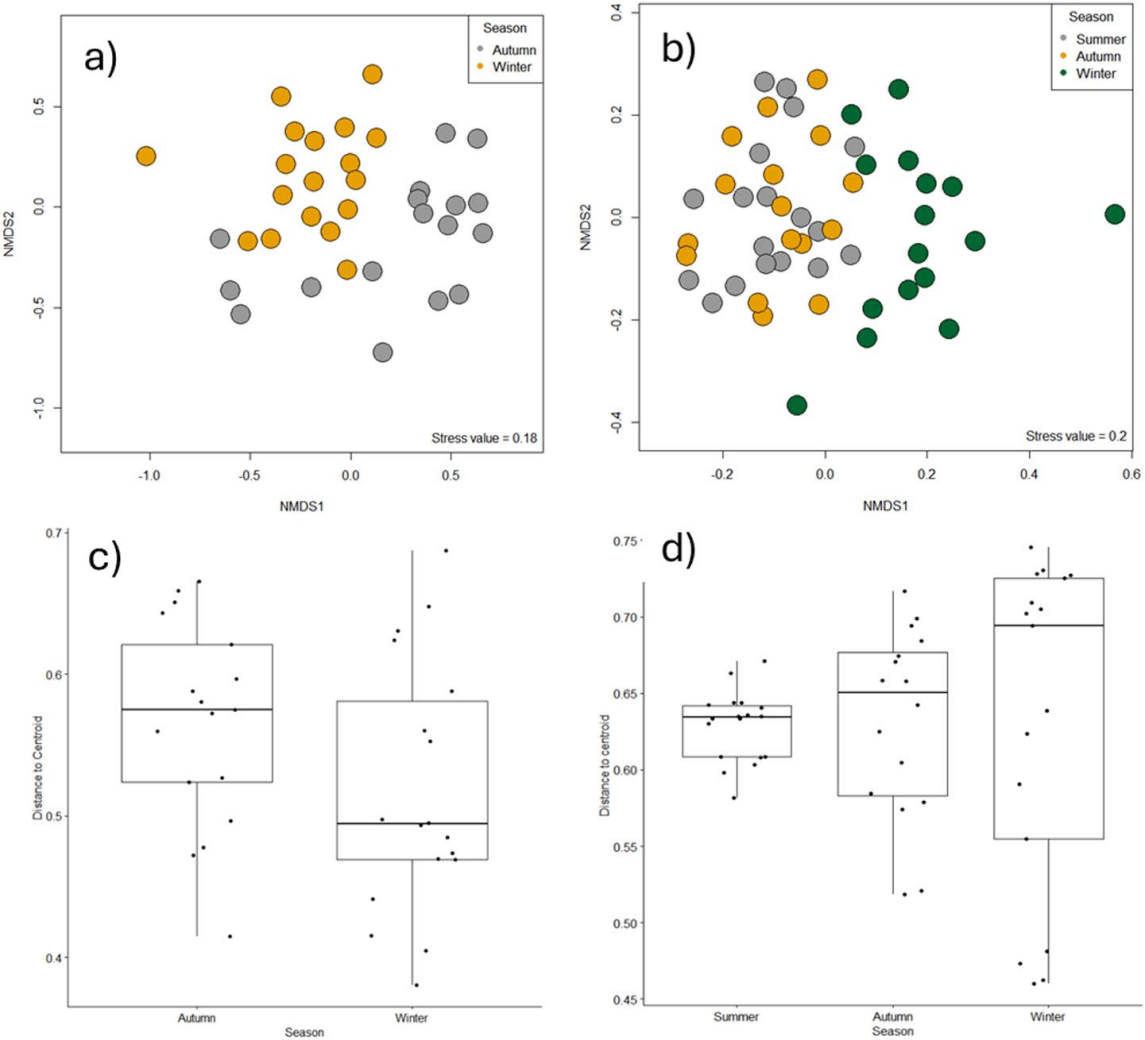
NMDS plots of dissimilarity in seasonal (**a**) stream benthic diatom communities and (**b**) pond phytoplankton communities (Bray Curtis dissimilarity): and boxplots of multivariate dispersion distances for autumn and winter benthic diatom communities from (**c**) urban streams and (**d**) ponds

PERMANOVA demonstrated that there was a significant difference in benthic diatom communities in the urban streams between the autumn and winter season (R^2^ = 0.105, *p* < 0.001), which is shown by the clear separation of seasons in the NMDS biplot (Fig. 3a). Multivariate dispersion of benthic diatom communities was not significantly different (F = 3.111, *p* = 0.087) between autumn (distance to centroid: 0.566) and winter among urban streams (distance to centroid: 0.517, Fig. 3b). A total of 48 species were only recorded from the autumn season, while 35 taxa were recorded only from the winter season (with 73 species recorded from both seasons: Supplementary Information Table S4) among stream sites.

Among pond sites, winter phytoplankton communities were significantly different to summer and autumn communities (pairwise PERMANOVA, *P* < 0.01, see Supplementary Information Table S5, for full results), which is shown by the clear seasonal separation in the NMDS biplot (Fig. 3b). The multivariate dispersion of phytoplankton communities was not significantly different (F = 0.013, *p* = 0.98) between summer (distance to centroid = 0.629), autumn (distance to centroid = 0.632) and winter (distance to centroid = 0.633) seasons (Fig. 3d). *Rhoicosphenia sp.* was recorded only from the winter season, while 12 phytoplankton taxa were unique to the summer and eight to the autumn seasons (Supplementary Information Table S6).

**Fig. 4:**
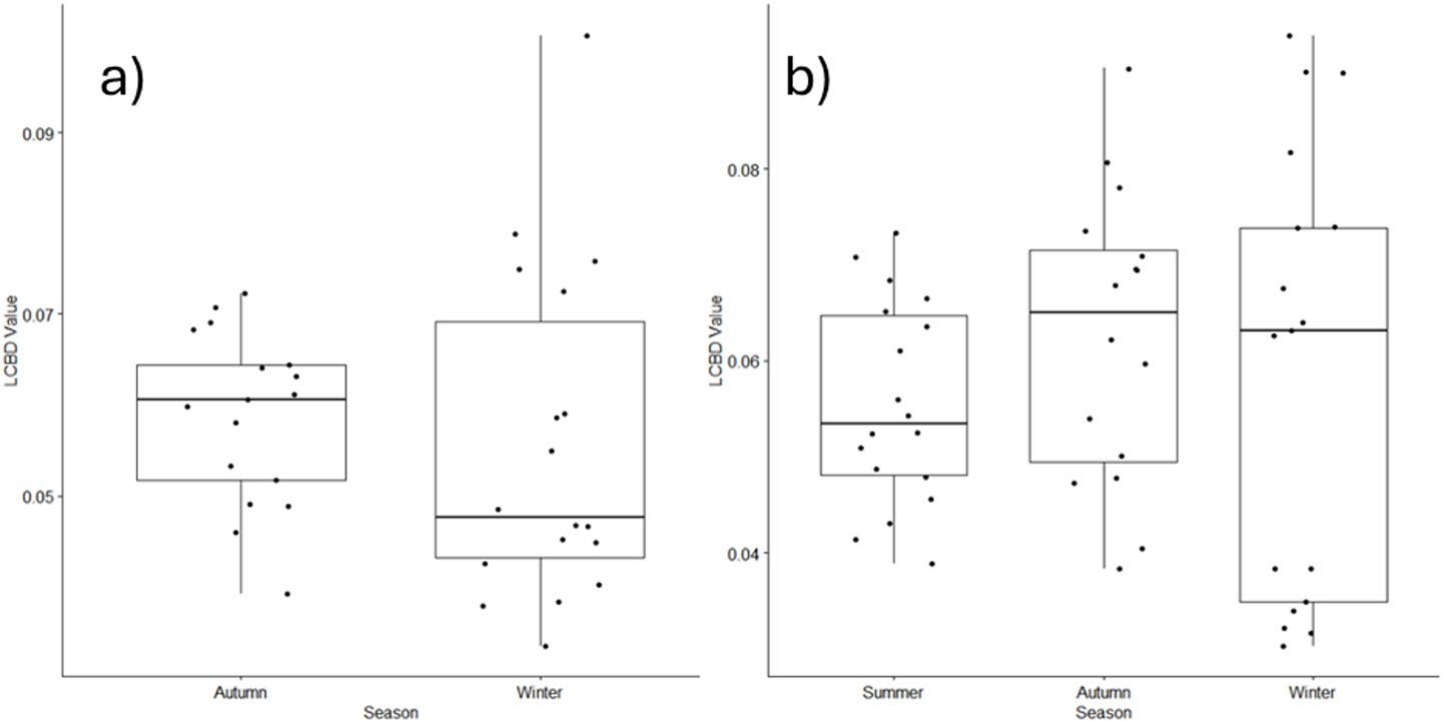
Median LCBD values recorded among (**a**) the diatom communities from the urban streams and (**b**) phytoplankton communities from the urban ponds, across the summer, autumn and winter seasons

Among stream samples, total beta diversity was higher among the benthic diatom communities during the autumn (0.41) than the winter (0.38), and for both seasons. Species replacement explained the majority of the variation (autumn: 85%, winter: 76%) compared to nestedness (autumn:15%, winter: 24%). Across the winter stream samples, three sites recorded significant LCBD values, whilst only one site recorded significant LCBD values in autumn. No significant difference in stream diatom LCBD values between autumn (median = 0.061) and winter (median = 0.048) seasons were recorded (Kruskal-Wallis Chi^2^ = 1.573, df = 1, *p* = 0.2098; Fig. 4a).

When urban ponds were considered, total beta diversity was comparable across the three seasons (summer: 0.39, autumn: 0.39, winter: 0.40). Species turnover dominated summer (turnover: 56%, nestedness: 44%) and autumn (turnover: 68%, turnover: 32%), whilst richness differences explained more of the compositional variation in winter (turnover: 48%, nestedness: 52%). Across each of the autumn and winter seasons, a total of 2 sites recorded significant LCBD values, whilst only one site in summer recorded significant LCBD values. No significant difference in pond phytoplankton LCBD values between summer (median = 0.053), autumn (median = 0.065) and winter (median = 0.063) seasons was recorded (Kruskal-Wallis Chi^2^ = 1.446 *df* = 2, *p* = 0.485; Fig. 4b).

### Species-environment relationships among urban benthic diatom stream and phytoplankton pond communities

Redundancy analysis of stream benthic diatom assemblages indicated seasonal differences in the associated variables (Table 3). Nitrate (NO_3_-N) concentrations were the only significant term in a weak but significant RDA (Adj. R^2^ 0.06, *P* < 0.05) using the autumn samples only. Winter diatom assemblages, however, were associated with the evidence of outflows (e.g., combined sewer overflows) and to a lesser extent, the alkalinity, ammonium (NH_4_-N), zinc and potassium concentrations in a highly significant model (Adj. R^2^ 0.25, *P* < 0.01). When combined autumn and winter samples, only iron concentrations were included as a significant term in a weak but significant model (Adj. R^2^ 0.05, *P* < 0.05). No significant model was established for phytoplankton in ponds in either the autumn or winter samples. However, pH was a significant term in both the summer analysis and when pooling data from all seasons. In the summer, pH was a highly significant term in a highly significant model (Adj. R^2^ 0.22, *P* < 0.01), similar to a marginally stronger model for all seasons combined (Adj. R^2^ 0.25, *P* < 0.01).

**Table 3:** RDA model summaries for benthic diatom and phytoplankton communities and environmental parameters. Parameters listed in final models are significant terms (*P* < 0.05). No model resolved for phytoplankton assemblages in autumn or winter

Model	Season	*R* ^2^	Adj. *R*^2^	Model *P*	RDA1	RDA2	RDA3	RDA4	RDA5
Benthic diatoms ~ NO3-N	Autumn	0.12	0.06	0.027	1.00	NA	NA	NA	NA
Benthic diatoms ~ Visual P (F2.5) + Alk (F2.1)+ NH4-N (F2.0) + Zn (F1.8) + K (F1.8)	Winter	0.47	0.25	0.001	0.35	0.25	0.20	0.11	0.10
Benthic diatoms ~ Fe (F1.8)	All sampled	0.10	0.05	0.012	0.57	NA	NA	NA	NA
Phytoplankton ~ pH (F5.76)	Summer	0.26	0.22	0.001	1.00	NA	NA	NA	NA
Phytoplankton ~ pH (F6.68)	All sampled	0.29	0.25	0.001	1.00	NA	NA	NA	NA

## Discussion

This study used a multi-ecosystem approach to investigate stream benthic diatom assemblages and pond phytoplankton communities across an urban landscape and seasons (Bristol, UK). Moreover, this study on phytoplankton diversity and community structure in ponds responds to existing knowledge gaps in this area, as indicated by Hill et al. (2021).

### Seasonal differences in taxonomic richness

Water quality analyses across both stream and pond sites revealed consistent eutrophic conditions, spanning mesotrophic to hypereutrophic states (see Supplementary Information Table S7), with nutrient concentrations, particularly nitrate and phosphorus, showing distinct seasonal variability. *Concomitantly*, taxonomic richness of diatom and phytoplankton assemblages displayed pronounced seasonal patterns, with both stream and pond sites exhibiting reduced richness during the winter months compared to autumn.

Nitrate emerged as the primary environmental variable influencing diatom diversity during autumn, a pattern consistent with previous findings that nitrogen availability can drive benthic algal productivity (Dodds 2006). This seasonal association is further supported by Liang et al. (2020), who documented seasonal changes in diatom assemblages in response to fluctuating nutrient levels in urban lakes. Likewise, Gabel et al. (2012) observed significant seasonal shifts in diatom communities in streams impacted by agricultural runoff, where nitrate concentrations varied seasonally due to land use and hydrological changes. Together, these findings suggest the importance of seasonal nutrient dynamics in shaping freshwater algal communities, and highlight differential responses in lentic versus lotic systems.

### Environmental influences on benthic diatom assemblages

Environmental observations at stream sites revealed the water eutrophic trophic condition (meso, eutrophic and hypereutrophic, see Supplementary Information Table S7), with seasonal fluctuations in phosphorus and nitrate concentrations. In evaluating the environmental variables that influenced diatom diversity and community structure, nitrate emerged as the primary factor during the autumn. This finding aligns with other published research showing that nitrogen alone, as well as the combination of nitrogen and phosphorus, are key drivers of benthic algae growth in both lentic and lotic systems, regardless of the season (Dodds 2006).

Significant seasonal differences were found between autumn and winter at the stream sites. Temperature and flow velocity might only indicate the natural changes in seasonal temperature changes and subsequent increase in rainfall during winter months. However, Teittinen et al. (2015) and Walker and Pan (2006) also identified temperature as a strong variable to influence diatom assemblages in urbanized regions compared to rural settings. Other variables that differed significantly between autumn and winter were pH, TSS, Al, Fe and Pb, indicating that winter storms or seasonal rainfall may change water clarity and could increase or decrease metal concentration with more or less precipitation as suggested by Newall and Walsh (2005). Similarly, NO_3_-N and PO_4_-P may have flushed out from storm ponds or accumulated in autumn. A strongly significant contribution came from the combined effects of pH, ammonium, zinc, and potassium in differentiating diatom communities among various stream sites for the winter assemblages. We found species that are frequently described as highly tolerant to anthropogenic pollutants in both seasons and across most of our sites: *Achnanthidium minutissimum*, *Amphora pediculus*, *Diatoma vulgaris*, *Gomphonema parvulum*, *Navicula gregaria*, *Navicula lanceolata*, *Reimeria sinuata*, *Rhoicosphenia abbreviata*, *Surirella brebisonii* (Teittinen et al., 2015; Morin et al., 2014 Walker and Pan 2006; Newall and Walsh 2005; Jüttner et al., 2012). We also found *Cocconeis euglypta*, *C. lineata* and *C. placentula* in both seasons, but contrary to Morin et al. (2014), who found these species in the least contaminated sites, at least for zinc. Interestingly, Jüttner et al. (2012) found *A. minutissimum* and *C. placentula* in sites with good or moderate ecological status but indicated that these genera have several morphotypes with a wide range of diverse habitat preferences, creating taxonomic challenges for their correct identification.

We observed that a small number of benthic diatom species accounted for ~ 10% of the total individual count across both seasons, indicating that, in many cases, only one individual was recorded at each site. Two species, *Psammodyctum panduriforme* and *Actinoptychus undulatus*, typically associated with marine environments (Guiry and Guiry, 2018), were found at a single site (RTCR) in the lower reaches of the River Trym near the tidal River Avon. The Avon experiences the second-highest tide in the world at 14 m, and the influence of the estuarine waters can lead to rapid daily changes in salinity, shaping the distribution of aquatic organisms and affecting habitat suitability for various species.

McGowan et al. (2020) concluded that benthic processes mediate water chemistry (changes in N forms and bioavailable P mobilisation) and that these indirect effects drive the phytoplankton compositional shifts in shallow lakes. Multiple studies have further identified species-specific responses in the algal community to nitrogen concentrations (Moss 1972; Dodds 2006; McGowan et al. 2020), contributing to the variation in community composition.

### Environmental influences on phytoplankton assemblages

As a result of their shallowness, all phytoplankton species in the water column can access light and therefore there is no competition for this resource, nor are they competing for nutrients in such eutrophic conditions (Reynolds et al., 1992). pH emerged as a significant factor in the summer analysis for phytoplankton communities in ponds. The pH level of the water can influence phytoplankton by affecting their metabolic processes and nutrient availability. High pH in the presence of cations such as Ca^2+^ or Mg^2+^ could lead to precipitates of phosphorus as compounds such as calcium phosphate, reducing the bioavailable fraction of phosphorus (Dillon and Molot 2024). Therefore, high pH levels can enhance the growth of certain phytoplankton species, leading to shifts in community composition. When data from all seasons were pooled, pH remained a significant term in the RDA, highlighting the importance of pH as a potential regulating factor for phytoplankton assemblages throughout the year. High primary productivity in ponds, resulting in abundant phytoplankton, leads to dynamic pH fluctuations. These changes are governed by the metabolic activity of phytoplankton, which influences the pH-carbon dioxide-bicarbonate equilibrium through diurnal photosynthetic uptake and nocturnal respiratory release of carbon dioxide (Brönmark and Hansson, 2017; Stumm and Morgan, 2013).

Alternatively, Moss (1972) demonstrated that high pH in alkaline, hard-water habitats restricts free CO_2_ availability, thereby limiting the growth of many phytoplankton species. In contrast, eutrophic species can utilize bicarbonate or thrive under very low CO_2_ concentrations, conferring a competitive advantage in these conditions. By comparison, species unable to use bicarbonate are disadvantaged in hard waters with low free CO_2_. As pH increases above ~ 8, the proportion of total inorganic carbon present as free CO₂ (CO₂(aq)) drops sharply; in hard-water lakes with high bicarbonate, pH often stabilizes above 8 (Moss, 1972). Although neither pH nor bicarbonate alone fully explains species distributions; the balance of inorganic carbon forms is critical. Temporary fluctuations in pH (e.g., daytime photosynthetic peaks or nighttime sinking of flagellates to low-pH layers) can allow certain algae to survive or grow in otherwise unfavourable layers (Moss, 1972). Under these conditions, we found several taxa known to be tolerant to high pH and low CO2 concentration, such as *Melosira* spp., *Asterionella* spp., *Fragilaria* spp., *Microcystis aeruginosa* and *Ceratium hirundinella*, which had been described in the literature (Reynolds 1984). For example, we found that *Eudorina* spp. and *Volvox* spp., which were only present in the summer, can become inhibited when the pH exceeds 9.5, but both *Microcystis* spp. and *Dolichospermum* spp. can continue to grow above this level. Reynolds (1984) and, more recently, Borics et al. (2021) describe the succession patterns of phytoplankton species in shallow lakes and ponds. Both studies highlight the complex dynamics of competition for limited resources, which gradually become scarce for certain phytoplankton species while being utilized by others. For example, we found that during summer, *Eudorina*, which Reynolds (1984) describes as thriving in high light availability, is outcompeted by *Scenedesmus*, *Pediastrum*, and *Coelastrum*, which appear later in the summer but are present in several seasons. We observed *Oscillatoria* exclusively during the summer and *Sphaerocystis* across multiple seasons, which contradicts the findings of Reynolds (1984).

No significant species-environment model was resolved for phytoplankton in ponds during either autumn or winter, suggesting that the environmental variables considered in the RDA might not be the primary factors influencing phytoplankton assemblages during these seasons. Phytoplankton dynamics could be driven by factors such as predation pressure, or interactions with other microorganisms. Borics et al. (2021) addressed the mechanisms driving freshwater phytoplankton diversity and their ecological implications. This highlights the dynamic interplay between equilibrium and non-equilibrium forces in shaping species diversity, emphasizing how disturbances, competition, and environmental gradients contribute to community assembly.

### Turnover in algal species composition between lentic and lotic ecosystems

The observed higher total beta-diversity of benthic diatoms in autumn (0.41) compared to winter (0.38) suggests that environmental conditions during autumn allow for slightly greater compositional turnover among urban streams. This seasonal distinction may reflect shifts in factors such as nutrient availability, flow regimes, or light penetration, which can vary markedly between autumn (e.g., leaf litter inputs, reduced canopy cover) and winter (e.g., lower temperatures, altered flow due to precipitation or snowmelt). In both seasons, turnover was the primary factor influencing the composition of benthic diatom assemblages, accounting for 85% of the variation in autumn and 76% in winter. This suggests that the differences in species present at each site, rather than the total number of species (nestedness), contributed significantly to the variations observed. This pattern indicates that each site may offer unique ecological niches or experience local environmental conditions that favour distinct diatom taxa. Additionally, it highlights the importance of spatial heterogeneity, where small-scale habitat differences—such as variations in substrate type, hydrological conditions, or site-specific pollution inputs—drive changes in community composition (Bolgovics et al. 2019).

The LCBD patterns provide additional insight into which stream sites have particularly unique diatom communities. While three sites showed significant LCBD values during winter, only one did so in autumn. Of particular interest were two sites of the Malago River (MM, MLNR), which presented the highest phosphorus concentrations and harboured five (~ 36%) of the 14 unique species found in seven sites of the 18 sampled. These findings are consistent with previous research indicating that phosphorus enriched habitats can support distinctive species assemblages (Biggs et al., 2017) but in contrast to the findings by Jüttner et al. (2012), where the most polluted sites only counted 2–3 taxa described in their study. Possible explanations for individual sites displaying a higher LCBD in winter include local events (e.g., pulses of road salt, temperature extremes, localized disturbance) that selectively favour certain diatom species while excluding others.

## Conclusions

Diatoms and phytoplankton are fundamental to primary productivity and nutrient cycling in aquatic environments. They are largely examined as indicators of water quality but must also be considered as an integral part of the ecosystem and be incorporated within urban conservation and management plans. Ponds are underrepresented in research and conservation policy compared to larger freshwater systems like lakes and large rivers, particularly within urban landscapes (Biggs et al. 2017; Hill et al. 2021).

Our study pioneers research in diversity for small water bodies (streams and ponds). We believe that with this study, we are contributing to filling this gap in research and encouraging other researchers to conduct further studies on the urban biodiversity of streams and ponds, and to increase understanding of the contribution of diatom and phytoplankton diversity in these water bodies, not just as bioindicators of water quality.

By broadening our view to include their subtle yet transformative ecological contributions, we can fortify existing initiatives and pave the way for more holistic, resilient approaches to preserving water ecosystems in an era of accelerating environmental change.

### Implications for conservation and management

- Algae are rarely considered in ecological conservation or management plans, but are an integral part of the ecosystem.
- Importance of nitrogen (N) in streams as a driver in benthic diatom assemblages, which indicates the need to protect streams with adequate vegetation to mitigate the effects of pollution sources.
- The man-made, shallow nature (< 3 m) of most of the ponds studied here suggests their utility as field-mesocosms or model systems (de Meester et al. 2005), where some are adjacent to the land (hence receiving groundwater ingress) or separated from the land (in the case of concrete ponds) and connected to the environment by their water supply.
- Materials used for pond construction should be reviewed, aiming for better integration into their natural environment and reducing their heating capacity, preventing the temperature increase from aggravating.

### Research gaps

Future research should consider exploring additional sampling across all seasons and environmental variables, and their interactions, to build more comprehensive models. Investigations into the role of light availability, temperature fluctuations, and biotic interactions could provide a deeper understanding of the complex dynamics governing diatom and phytoplankton communities, and their resilience to environmental change. Moreover, long-term monitoring studies could help capture temporal variations and trends that might be missed in short-term analyses. Addressing these gaps is vital for the sustainable management of pond ecosystems and for reversing the decline in freshwater biodiversity globally. Furthermore, socio-economic considerations, including the contribution of ponds and streams to human well-being and their potential as educational and recreational resources cannot be ignored for the adoption of conservation measures in such social-ecological systems (Thornhill et al., 2022).

Thus, in general, studies on phytoplankton have identified relatively low alpha diversity in small water bodies while revealing metacommunities harbouring unique microflora of significant conservation value (Bolgovics et al. 2019). Further research into the algal diversity of small water bodies, among the most endangered freshwater habitats (Borics et al., 2021), is crucial for improving our understanding of algal diversity and the conservation value of small water bodies. Our understanding of phytoplankton and benthic diatom biodiversity remains incomplete. While research on “small water bodies” exists to some degree, this category is not synonymous with “urban water body”, and the unique ecological pressure within urban environments needs targeted research. This knowledge gap hinders the effective conservation of urban aquatic ecosystems.

## Supplementary Information

Below is the link to the electronic supplementary material.

Supplementary Material_Tables S1-S8

## Data Availability

The datasets generated during and/or analysed during the current study are available from the corresponding author on reasonable request.

## References

[CR1] Abonyi A, Acs E, Hidas A, Grigorszky I, Várbíró G, Borics G, Kiss KT (2018) Functional diversity of phytoplankton highlights long-term gradual regime shift in the middle section of the Danube River due to global warming, human impacts and oligotrophication. Freshw Biol 63(5):456–472

[CR96] Biggs J, Von Fumetti S, Kelly-Quinn M (2017) The importance of small waterbodies for biodiversity and ecosystem services: implications for policy makers. Hydrobiol 793(1):3–39

[CR5] Bishop I, Boldrini A, Clymans W, Hall C, Moorhouse H, Parkinson S, Scott-Somme K, Thornhill I, Loiselle S (2025) FreshWater Watch: Investigating the Health of Freshwater Ecosystems, from the Bottom Up. Citizen Science: Theory and Practice, 10(1)

[CR101] Blanco S, Bécares E, Cauchie HM, Hoffmann L, Ector L (2007) Comparison of biotic indices for water quality diagnosis in the Duero Basin (Spain). Large Riv 267–286

[CR6] Bolgovics Á, Viktória B, Várbíró G, Krasznai-K EÁ, Ács É, Kiss KT, Borics G (2019) Groups of small lakes maintain larger microalgal diversity than large ones. Sci Total Environ 678:162–172 31075582 10.1016/j.scitotenv.2019.04.309

[CR97] Borics G, Abonyi A, Salmaso N, Ptacnik R (2021) Freshwater phytoplankton diversity: models, drivers and implications for ecosystem properties. Hydrobiol 848(1):53–75. 10.1007/s10750-020-04332-9 PMC7334633 32836348

[CR89] Brierley B, Carvalho L, Davies S, Krokowski J (2007) Guidance on the quantitative analysis of phytoplankton in Freshwater Samples. Report to SNIFFER (Project WFD80), Edinburgh, December 2007

[CR7] Brierley B, Carvalho L, Davies S, Krokowski J (2007) Guidance on the quantitative analysis of phytoplankton in Freshwater Samples. Report to SNIFFER (Project WFD80), Edinburgh, December 2007

[CR8] Brönmark C, Hansson LA (2017) The biology of lakes and ponds. Oxford University Press

[CR9] Carter CF, John DM, Wilbraham J (2016) AlgaeVision: Virtual Collection of Freshwater Algae from the British Isles. Version II. World Wide Web electronic publication. www.nhm.ac.uk/algaevision.html

[CR71] Castrignanò E, Yang Z, Feil EJ, Bade R, Castiglioni S, Causanilles A, Gracia-Lor E, Hernandez F, Plósz BG, Ramin P, Rousis NI (2020) Enantiomeric profiling of quinolones and quinolones resistance gene qnrS in European wastewaters. Water res 175:115653. 10.1016/j.watres.2020.115653 32208173

[CR72] Castro-Castellon AT, Horton AA, Hughes JM, Rampley C, Jeffers ES, Bussi G, Whitehead P (2022) Ecotoxicity of microplastics to freshwater biota: Considering exposure and hazard across trophic levels. Sci Total Environ 816:151638. 10.1016/j.scitotenv.2021.151638 34774956

[CR77] Cemagref C (1982) Étude des méthodes biologiques quantitative d’appréciation de la qualité des eaux. Rapport Division Qualité des Eaux Lyon—Agence financière de Bassin Rhône-Méditerranée-Corse: Pierre-Bénite 218.

[CR70] Davies B, Biggs J, Williams P, Whitfield M, Nicolet P, Sear D, Bray S, Maund S (2008) Comparative biodiversity of aquatic habitats in the European agricultural landscape. Agriculture, Ecosys Environ 125(1-4):1–8.

[CR11] De Meester L, Declerck S, Stoks R, Louette G, Van De Meutter F, De Bie T, Michels E, Brendonck L (2005) Ponds and pools as model systems in conservation biology, ecology and evolutionary biology. Aquat conservation: Mar Freshw Ecosyst 15(6):715–725

[CR12] Dillon PJ, Molot LA (2024) The Phosphorus Cycle. In Wetzel’s Limnology (pp. 359–425). Edited by Jones, I.D. and Smol, J.P. Academic Press

[CR13] Dodds WK (2006) Eutrophication and trophic state in rivers and streams. Limnol Oceanogr 51(1 part 2):671–680

[CR14] Downing JA, Watson SB, McCauley E (2001) Predicting cyanobacteria dominance in lakes. Can J Fish Aquat Sci 58(10):1905–1908

[CR15] Dray S, Bauman D, Blanchet G, Borcard D, Clappe S, Guenard G, Jombart T, Larocque G, Legendre P, Madi N, Wagner HH, Siberchicot A (2024) adespatial: Multivariate multiscale spatial analysis. R package version 0.3–24. [Accessible at: https://cran.r-project.org/web/packages/adespatial/adespatial.pdf]

[CR16] Environment Agency https://environment.data.gov.uk/catchment-planning/OperationalCatchment/3046)

[CR74] Field CB, Behrenfeld MJ, Randerson JT, Falkowski P (1998). Primary production of the biosphere: integrating terrestrial and oceanic components. sci 281(5374):237–240. 10.1126/science.281.5374.237 9657713

[CR17] Gabel KW, Wehr JD, Truhn KM (2012) Assessment of the effectiveness of best management practices for streams draining agricultural landscapes using diatoms and macroinvertebrates. Hydrobiologia 680:247–264

[CR80] González-Paz L, Delgado C, Pardo I (2020) Understanding divergences between ecological status classification systems based on diatoms. Sci Total Environ 734:139418. 10.1016/j.scitotenv.2020.139418 32460081

[CR100] Guiry MD, Guiry GM (2024) AlgaeBase. World-wide electronic publication, University of Galway. https://www.algaebase.org

[CR19] Guiry MD in Guiry MD, Guiry GM (eds) 12 November 2018. AlgaeBase. World-wide electronic publication, National University of Ireland, Galway. ; online access to database searched on various occasions, last entry 14 January 2025 https://www.algaebase.org

[CR21] Heino J, Melo AS, Siqueira T, Soininen J, Valanko S, Bini LM (2015) Metacommunity organisation, spatial extent and dispersal in aquatic systems: patterns, processes and prospects. Freshw Biol 60:845–869

[CR87] Heino J, Soininen J, Alahuhta J, Lappalainen J, Virtanen R (2017) Metacommunity ecology meets biogeography: effects of geographical region, spatial dynamics and environmental filtering on community structure in aquatic organisms. Oecologia 183(1):121-137. 10.1007/s00442-016-3750-y 27714463

[CR86] Hill MJ, Greaves HM, Sayer CD, Hassall C, Milin M, Milner VS, Marazzi L, Hall R, Harper LR, Thornhill I, Walton R (2021) Pond ecology and conservation: research priorities and knowledge gaps. Ecosphere 12(12):e03853.

[CR110] Hill MJ, Thornhill I, Tiegs SD, Castro-Castellon A, Hernández-Avilés JS, Daw A, Salinas-Camarillo VH, Hobbs S (2022) Organic-matter decomposition in urban stream and pond habitats. Ecol Indicat 142:109232.

[CR22] Hill MJ, Thornhill I, Briers RA, Gledhill DG, White JC, Wood PJ, Hassall C (2017) Urban ponds as an aquatic biodiversity resource in modified landscapes. Glob Change Biol 23(3):986–999 10.1111/gcb.13401 27476680

[CR23] Jeppesen E, Søndergaard M, Jensen JP, Havens KE, Anneville O, Carvalho L, Coveney MF, Deneke R, Dokulil MT, Foy BOB, Gerdeaux D (2005) Lake responses to reduced nutrient loading–an analysis of contemporary long-term data from 35 case studies. Freshw Biol 50(10):1747–1771

[CR24] John DM, Whitton BA, Brook AJ (eds) (2011) The freshwater algal flora of the British Isles: An identification guide to freshwater and terrestrial algae. Cambridge University Press

[CR83] Jüttner I, Chimonides PJ, Ormerod SJ (2012) Developing a diatom monitoring network in an urban river-basin: initial assessment and site selection. Hydrobiol 695(1):137–151.

[CR26] Kahlert M, Kelly M, Albert RL, Almeida SF, Bešta T, Blanco S, Coste M, Denys L, Ector L, Fránková M, Hlúbiková D (2012) Identification versus counting protocols as sources of uncertainty in diatom-based ecological status assessments, vol 695. Hydrobiologia, pp 109–124

[CR28] Kelly MG, Whitton BA (1995) The trophic diatom index: a new index for monitoring eutrophication in rivers. J Appl Phycol 7:433–444 K

[CR27] Kelly M, King L, Chatháin BN (2009) the conceptual basis of ecological—status assessments using diatoms. In biology and environment: proceedings of the Royal Irish Academy (Vol. 109, No. 3, pp. 175–189). Royal Irish Academy

[CR30] Lange-Bertalot H, Hofmann G, Werum M, Cantonati M (2017) Freshwater benthic diatoms of Central Europe: Over 800 common species used in ecological assessment. Edited by Martyn G. Kelly. Vol. 942. Schmitten-Oberreifenberg: Koeltz Botanical Books

[CR90] Legendre P, De Cáceres M (2013) Beta diversity as the variance of community data: dissimilarity coefficients and partitioning. Ecol lett 16(8):951–963 10.1111/ele.12141 23809147

[CR32] Liang J, Huang C, Stevenson MA, Qiao Q, Zeng L, Chen X (2020) Changes in summer diatom composition and water quality in urban lakes within a metropolitan area in central China. Int Rev Hydrobiol 105(3–4):94–105

[CR34] Maberly SC, O’Donnell RA, Woolway RI, Cutler ME, Gong M, Jones ID, Merchant CJ, Miller CA, Politi E, Scott EM, Thackeray SJ (2020a) Global lake thermal regions shift under climate change. Nature communications, 11(1), p.1232 10.1038/s41467-020-15108-z PMC7060244 32144247

[CR35] Maberly SC, Pitt JA, Davies PS, Carvalho L (2020b) Nitrogen and phosphorus limitation and the management of small productive lakes. Inland Waters 10(2):159–172

[CR36] Maloufi S, Catherine A, Mouillot D, Louvard C, Couté A, Bernard C, Troussellier M (2016) Environmental heterogeneity among lakes promotes hyper β-diversity across phytoplankton communities. Freshw Biol 61(5):633–645

[CR73] Mann DG (1999) The species concept in diatoms. Phycol 38(6):437–495.

[CR37] Mann DG, Vanormelingen P (2013) An inordinate fondness? The number, distributions, and origins of diatom species. J Eukaryot Microbiol 60(4):414–420 23710621 10.1111/jeu.12047

[CR38] McDonnell MJ, Hahs AK (2008) The use of gradient analysis studies in advancing our understanding of the ecology of urbanizing landscapes: current status and future directions, vol 23. Landscape Ecology, pp 1143–1155

[CR39] McGowan S, Leavitt PR, Barker T, Moss B (2020) Shallow water phytoplankton responses to nitrate and salinity enrichment may be modified by benthic processes. Inland Waters 10(1):137–151

[CR41] Morton RD, Marston CG, O’Neil AW, Rowland CS (2021) Land Cover Map 2020 (25m rasterised land parcels, GB). NERC EDS Environ Inform Data Centre. 10.5285/6c22cf6e-b224-414e-aa85-900325baedbd

[CR94] Moss AJ (1972) Bed‐load sediments. Sedimentol 18(3–4):159–219.

[CR42] Newall P, Walsh CJ (2005) Response of epilithic diatom assemblages to urbanization in f luences. Hydrobiologia 532:53–67

[CR43] Oertli B, Parris KM (2019) Toward management of urban ponds for freshwater biodiversity. Ecosphere 10(7):e02810

[CR44] Oksanen J, Simpson GL, Blanchet FG, Kindt R, Legendre P, Minchin PR, O’hara RB, Solymos P, Stevens MHH, Szoecs E, Wagner H, Barbour M, Bedward M, Bolker B, Borcard D, Carvalho G, Chirico M, De Caceres M, Durand S, Evangelista HBA, FitzJohn R, Friendly M, Furneaux B, Hannigan G, Hill MO, Lahti L, McGlinn D, Ouellette M, Cunha ER, Smith T, Stier A, Ter Braak CJF, Weedon J, Borman T (2025) vegan: *Community* ecology package. R Package Version 2.6–10. [Accessible at: https://cran.r-project.org/web/packages/vegan/vegan.pdf]

[CR47] Paerl HW, Huisman J (2008) Blooms like it hot. Science 320(5872):57–58 18388279 10.1126/science.1155398

[CR46] Paul MJ, Meyer JL (2001) Streams in the urban landscape. Annu Rev Ecol Syst 32(1):333–365

[CR48] Pohlert 2024 https://cran.r-project.org/web/packages/PMCMRplus/PMCMRplus.pdf

[CR76] Prygiel J, Coste M, Bukowska JOANNA (1999) Principales techniques d'évaluation de la qualité des rivières basées sur les diatomées-Etat de l'art en Europe.

[CR79] Ptacnik R, Andersen T Tamminen T (2010) Performance of the Redfield ratio and a family of nutrient limitation indicators as thresholds for phytoplankton N vs. P limitation. Ecosys 13(8):1201–1214

[CR75] Ptacnik R, Solimini AG, Andersen T, Tamminen T, Brettum P, Lepistö L, Willén E, Rekolainen S (2008) Diversity predicts stability and resource use efficiency in natural phytoplankton communities. Proc Natl Acad Sci 105(13):5134–5138 10.1073/pnas.0708328105 PMC2278227 18375765

[CR49] Reid AJ, Carlson AK, Creed IF, Eliason EJ, Gell PA, Johnson PT, Kidd KA, MacCormack TJ, Olden JD, Ormerod SJ, Smol JP (2019) Emerging threats and persistent conservation challenges for freshwater biodiversity. Biol Rev 94(3):849–873 30467930 10.1111/brv.12480

[CR95] Reynolds CS (1984) Phytoplankton periodicity: the interactions of form, function and environmental variability. Freshwater biol 14(2):111–142

[CR92] Reynolds CS, Bellinger EG (1992) Patterns of abundance and dominance of the phytoplankton of Rostherne Mere, England: evidence from an 18-year data set. Aqua Sci 54(1):10–36

[CR51] Rowan J, Duck S, Carwardine RW, Bragg J, Black OM, Cuttler AR, M, E.J., Souter I (2006) Lake Habitat Survey in the United Kingdom–Field Survey Guidance Manual, version 3.1. Final Report Project WFD. SNIFFER

[CR78] Salmaso N, Buzzi F, Cerasino L, Garibaldi L, Leoni B, Morabito G, Rogora M, Simona M (2014) Influence of atmospheric modes of variability on the limnological characteristics of large lakes south of the Alps: a new emerging paradigm. Hydrobiol 731(1):31–48.

[CR82] Sauer EL, Cruz J, Crone E, Lewis C, Plumier E, Cwynar B, Drake D, Herrick BM, Preston DL (2022) Multiscale drivers of amphibian community occupancy in urban ponds. Urban Ecosys 25(5):1469–1479.

[CR81] Sauer K, Stoodley P, Goeres DM, Hall-Stoodley L, Burmølle M, Stewart PS, Bjarnsholt T (2022) The biofilm life cycle: expanding the conceptual model of biofilm formation. Nat Rev Microbiol 20(10):608–620. 10.1038/s41579-022-00767-0 PMC9841534 35922483

[CR56] Spaulding SA, Potapova MG, Bishop IW, Lee SS, Gasperak TS, Jovanoska E, Furey PC, Edlund MB (2021) Diatoms. org: supporting taxonomists, connecting communities. Diatom Res 36(4):291–304. 10.1080/0269249X.2021.2006790 10.1080/0269249X.2021.2006790 PMC9359083 35958044

[CR93] Stumm W, Morgan JJ (2013) Aquatic chemistry: chemical equilibria and rates in natural waters. John Wiley & Sons.

[CR84] Teittinen A, Taka M, Ruth O, Soininen J (2015) Variation in stream diatom communities in relation to water quality and catchment variables in a boreal, urbanized region. Sci Total Environ 530:279-289. 10.1016/j.scitotenv.2015.05.101 26047862

[CR58] Thackeray SJ, Henrys PA, Hemming D, Bell JR, Botham MS, Burthe S, Helaouet P, Johns DG, Jones ID, Leech DI, Mackay EB (2016) Phenological sensitivity to climate across taxa and trophic levels. Nature 535(7611):241–245 27362222 10.1038/nature18608

[CR59] Thornhill I, Chautard A, Loiselle S (2018) Monitoring biological and chemical trends in temperate still waters using citizen science. Water 10(7):839

[CR60] Thornhill I, Hill MJ, Castro-Castellon A, Gurung H, Hobbs S, Pineda-Vazquez M, Gómez-Osorio MT, Hernández-Avilés JS, Novo P, Mesa-Jurado A, Calderon-Contreras R (2022) Blue-space availability, environmental quality and amenity use across contrasting socioeconomic contexts. Appl Geogr 144:102716

[CR85] Tornés E, Mor JR, Mandaric L, Sabater S (2018) Diatom responses to sewage inputs and hydrological alteration in Mediterranean streams. Environ Pollut 238:369–378. 10.1016/j.envpol.2018.03.037 29574361

[CR61] Tóth R, Czeglédi I, Kern B, Erős T (2019) Land use effects in riverscapes: Diversity and environmental drivers of stream fish communities in protected, agricultural and urban landscapes, vol 101. Ecological indicators, pp 742–748

[CR62] UKTAG 2022. UK Technical Advisory Group on the Water Framework Directive River Basin Management (2015–2021). www.wfduk.org Accessed online on 01.11.24 https://www.wfduk.org/sites/default/files/Media/UKTAG%20Phosphorus%20Standards%20for%20Rivers_Final%20130906_0.pdf

[CR64] Walker CE, Pan Y (2006) Using diatom assemblages to assess urban stream conditions.Advances in algal biology: A commemoration of the work of Rex Lowe, pp.179–189

[CR65] Walsh CJ, Sharpe AK, Breen PF, Sonneman JA (2001) Effects of urbanization on streams of the Melbourne region, Victoria, Australia. I. Benthic macroinvertebrate communities. Freshw Biol 46(4):535–551

[CR88] Wetzel CE, Lange-Bertalot H, Ector L (2017) Type analysis of Achnanthes oblongella Østrup and resurrection of Achnanthes saxonica Krasske (Bacillariophyta). Nova Hedwigia, Beihefte 146:209–227

[CR67] Winder M, Cloern JE (2010) The annual cycles of phytoplankton biomass. Phil Trans R Soc B 3653215–3653226. 10.1098/rstb.2010.0125 10.1098/rstb.2010.0125 PMC2981943 20819814

[CR68] Winder M, Sommer U (2012) Phytoplankton response to a changing climate. Hydrobiologia 698:5–16

