# Supplementary material for: Benthic diatoms and phytoplankton diversity in urban streams and ponds: charting a course for conservation
Source: Biodivers Conserv. 2026 Apr 17;35(6):149. doi: 10.1007/s10531-026-03305-z (PMC13090212; doi:10.1007/s10531-026-03305-z)
Supplement: Supplementary file 1 — Supplementary Material_Tables S1-S8 [file 10531_2026_3305_MOESM1_ESM.docx]

Supplementary Information

**Table S1.** Urban pond and stream Site name and Site ID used throughout the manuscript*.*

| Site ID | Pond system name | Site ID | Flow system name |
| --- | --- | --- | --- |
| AGP | Ashton Gate Pond 2 | BBWL | Brislington Brook West Town Lane |
| AOP | Ashton Court Ornamental | BSA | Brislington Brook at St. Annes Wood |
| BCP | Hazel Brook Pond - Blaise Castle | BWS | Baddocks Wood |
| BG | Uni. Bristol Botanical Gardens | CBR | Combe Brook at Royate Hill allotments |
| BHP | Brandon Hill Pond | CBWB | Combe Brook at Wooden Bridge |
| CPP | Canford Park pond | FEALS | R. Frome Eastville Park |
| CRP | Callington Rd. Nature Reserve Pond | HBCL | Hazel Brook - Crow Lane |
| CTW | Cabot Tower | HBHR | Hazel Brook - Henbury Road intersection |
| EVL | Eastville Park | HBMR | Horfield Brook Mina Road |
| HZL | Henleaze | MM | Malago Manor Wood Valley Rd. |
| LIDO | Lido | MRSD | Malago LNR |
| OBC | Oldbury Court | PHI | Pigeonhouse downstream Imperial Park |
| PHCB | Pigeonhouse Crox Bottom | PHUL | Pigeonhouse Stream, Far U/S |
| RFG | Royal Fort Gardens | RFBB | R. Frome, Bradley Brook |
| SDL | Sneyd Park | RFSM | R. Frome, Snuff Mills Begbroke School |
| SGP | St. George's Park | RTCD | R. Trym, Coombe Dingle Car Park |
| SKP | Stoke Park | RTCR | R. Trym, Trym Cross Road |
| SMT | St. Monica Trust | RTMC | R. Trym, Merlin Close |

**Table S2.** Rapid assessment metrics applied to urban ponds and streams. Broadleaved Woodland (BL WoodL), coniferous woodland (CF WoodL), arable horticulture (Arab Horticulture), improved grassland (Imp. GrasslL), urban, suburban (Suburb.), habitat complexity (Hab Comp.), colour, visual pollution (Visual Poll.), shade, fish.

| Site ID | Area m2 | Width m | BL WoodL % | CF WoodL % | Arab. Horti-culture % | Imp. GrassL % | Urban % | Suburb. % | Hab Comp. | Colour | Visual Poll. | Shade % | Fish |
| --- | --- | --- | --- | --- | --- | --- | --- | --- | --- | --- | --- | --- | --- |
| AGP | 151 | NA | 34.63 | 0.11 | 2.57 | 58.97 | 0.06 | 3.67 | 1.33 | 100.00 | 1.00 | 13.33 | 0 |
| AOP | 95 | NA | 31.42 | 0.11 | 1.58 | 61.44 | 0.47 | 4.99 | 4.00 | 33.33 | 0.00 | 43.33 | 0 |
| BBWL | NA | 2.80 | 6.91 | 0.00 | 1.60 | 14.59 | 4.74 | 72.16 | 2.33 | 0.00 | 0.50 | 82.50 | 0 |
| BCP | 912 | NA | 59.72 | 2.82 | 0.23 | 24.44 | 0.56 | 12.18 | 3.00 | 100.00 | 1.00 | 33.33 | 0 |
| BG | 146 | NA | 6.70 | 0.62 | 0.33 | 10.76 | 0.99 | 80.61 | 5.75 | 0.00 | 1.00 | 15.00 | 1 |
| BHP | 240 | NA | 6.59 | 0.00 | 0.11 | 3.08 | 27.46 | 60.11 | 3.75 | 33.33 | 0.00 | 57.50 | 0 |
| BSA | NA | 2.53 | 12.25 | 0.00 | 1.16 | 6.04 | 22.10 | 55.70 | 2.50 | 25.00 | 1.00 | 46.67 | 1 |
| BWS | NA | 3.00 | 14.94 | 0.20 | 0.06 | 7.64 | 2.11 | 74.36 | 2.25 | 0.00 | 0.67 | 72.50 | 0 |
| CBR | NA | 3.50 | 11.09 | 0.00 | 0.50 | 16.23 | 3.49 | 68.69 | 2.00 | 50.00 | 1.00 | 75.00 | 0 |
| CBWB | NA | 2.00 | 3.45 | 0.00 | 0.08 | 10.28 | 16.40 | 69.79 | 2.00 | 0.00 | 1.00 | 93.33 | 0 |
| CPP | 140 | NA | 10.08 | 0.01 | 2.26 | 19.47 | 1.32 | 66.87 | 1.33 | 100.00 | 1.00 | 15.00 | 1 |
| CRP | 141 | NA | 0.98 | 0.00 | 0.08 | 5.85 | 14.48 | 78.62 | 3.25 | 25.00 | 0.50 | 10.00 | 1 |
| CTW | 433 | NA | 10.48 | 0.00 | 16.55 | 34.60 | 3.27 | 35.11 | 3.00 | 60.00 | 1.00 | 40.00 | 1 |
| EVL | 12498 | NA | 24.29 | 0.10 | 2.33 | 24.80 | 4.90 | 42.08 | 2.25 | 100.00 | 1.33 | 30.00 | 1 |
| FEALS | NA | 12.75 | 16.51 | 0.00 | 1.71 | 22.61 | 11.68 | 46.59 | 1.67 | 100.00 | 0.67 | 66.67 | 0 |
| HBCL | NA | 3.57 | 3.55 | 0.00 | 3.95 | 20.80 | 5.03 | 66.68 | 2.75 | 25.00 | 1.00 | 80.00 | 0 |
| HBHR | NA | 2.73 | 13.93 | 0.11 | 1.88 | 27.24 | 4.08 | 52.76 | 1.00 | 75.00 | 0.75 | 75.00 | 0 |
| HBMR | NA | 1.15 | 8.99 | 0.00 | 2.08 | 8.19 | 6.80 | 73.93 | 4.00 | 0.00 | 0.67 | 80.00 | 0 |
| HZL | 13854 | NA | 13.46 | 0.16 | 0.04 | 11.98 | 3.40 | 70.28 | 6.25 | 100.00 | 1.00 | 15.00 | 1 |
| LIDO | 3646 | NA | 3.37 | 0.19 | 2.06 | 6.99 | 10.18 | 76.95 | 6.00 | 0.00 | 0.00 | 13.33 | 1 |
| MM | NA | 5.63 | 7.67 | 0.00 | 0.47 | 7.43 | 3.21 | 81.23 | 2.00 | 33.33 | 0.67 | 80.00 | 0 |
| MRSD | NA | 3.17 | 1.20 | 0.00 | 0.04 | 4.91 | 39.60 | 54.25 | 3.00 | 0.00 | 1.00 | 70.00 | 0 |
| OBC | 954 | NA | 38.35 | 1.09 | 0.62 | 24.67 | 0.07 | 35.14 | 4.67 | 33.33 | 1.50 | 73.33 | 0 |
| PHCB | 2062 | NA | 17.57 | 0.03 | 2.85 | 8.44 | 21.53 | 49.23 | 4.00 | 66.67 | 1.67 | 50.00 | 0 |
| PHI | NA | 4.80 | 19.52 | 0.00 | 0.68 | 13.45 | 9.23 | 57.11 | 2.75 | 25.00 | 1.50 | 70.00 | 0 |
| PHUL | NA | 2.15 | 3.55 | 0.00 | 4.64 | 6.36 | 13.47 | 71.99 | 2.00 | 0.00 | 1.00 | 65.00 | 0 |
| RFBB | NA | 10.00 | 17.19 | 0.00 | 3.62 | 45.04 | 2.09 | 32.06 | 2.50 | 66.67 | 1.00 | 63.33 | 1 |
| RFG | 149.5 | NA | 2.27 | 0.00 | 0.04 | 0.43 | 29.35 | 67.92 | 5.50 | 0.00 | 0.00 | 20.00 | 1 |
| RFSM | NA | 16.44 | 24.68 | 0.46 | 1.02 | 8.41 | 9.28 | 56.07 | 4.00 | 33.33 | 1.00 | 70.00 | 1 |
| RTCD | NA | 3.97 | 21.89 | 0.71 | 0.05 | 3.33 | 0.06 | 73.95 | 2.67 | 33.33 | 1.00 | 73.33 | 1 |
| RTCR | NA | 3.83 | 10.14 | 0.00 | 0.46 | 10.45 | 1.68 | 76.90 | 2.50 | 0.00 | 0.50 | 20.00 | 1 |
| RTMC | NA | 2.33 | 10.39 | 0.02 | 1.23 | 22.43 | 0.95 | 64.99 | 2.50 | 0.00 | 0.67 | 82.50 | 1 |
| SDL | 2510 | NA | 14.94 | 0.08 | 0.00 | 2.06 | 2.09 | 64.55 | 2.75 | 25.00 | 0.67 | 35.00 | 0 |
| SGP | 6305 | NA | 6.78 | 0.02 | 0.81 | 7.04 | 4.48 | 80.47 | 2.50 | 25.00 | 1.00 | 32.50 | 1 |
| SKP | 7813 | NA | 30.64 | 0.03 | 1.43 | 47.45 | 2.09 | 17.61 | 3.00 | 75.00 | 0.33 | 13.33 | 1 |
| SMT | 181 | NA | 2.42 | 0.00 | 0.19 | 4.97 | 2.03 | 90.38 | 3.75 | 100.00 | 0.50 | 17.50 | 1 |

Table S3. Covarying variables (Spearman’s Rank ≥ 0.80) and retained variable

| Model (system) | Season | Covariables | Removed |
| --- | --- | --- | --- |
| Phytoplankton (ponds) | All sampled (summer / autumn / winter) | Various ≈ Conductivity  Pb ≈ Fe  Emergent veg. ≈ Open water  Nitrate ≈ Woody debris  Urban ≈ Woodland | Conductivity  Fe  Open water  Woody debris  Woodland |
| Diatoms (rivers) | All sampled (autumn / winter) | Various ≈ Conductivity  K ≈ Mg | Conductivity  Mg |

**Table S4.** Benthic diatom taxa recorded uniquely from a single season (Autumn or Winter) and across both sample seasons in the urban streams.

| **Taxa** | **Autumn** | | **Winter** | |  |
| --- | --- | --- | --- | --- | --- |
| **Phylym** Heterokontophyta | |  | |  | |
| **Subphylum** Coscinodiscophytina | |  | |  | |
| **Class** Coscinodiscophyceae | |  | |  | |
| **Subclass** Coscinodiscophycidae | |  | |  | |
| **Order** Aulacoseirales | |  | |  | |
| **Family** Aulacoseiraceae | |  | |  | |
| **Genus** *Aulacoseira* | |  | |  | |
| *Aulacoseira granulata* (Ehrenberg) Simonsen 1979 | | X | |  | |
| *Aulacoseira* sp. | | X | | X | |
| **Order** Coscinodiscales | |  | |  | |
| **Family** Heliopeltaceae | |  | |  | |
| **Genus** *Actinoptychus* | |  | |  | |
| *Actinoptychus senarius* (Ehrenberg) Ehrenberg 1843 | | X | |  | |
| **Family** Hemidiscaceae | |  | |  | |
| **Genus** *Actinocyclus* | |  | |  | |
| *Actinocyclus normanii* (W. Gregory ex Greville) Hustedt 1957 | | X | | X | |
| **Subclass** Melosirophycidae | |  | |  | |
| **Order** Melosirales | |  | |  | |
| **Family** Melosiraceae | |  | |  | |
| **Genus** *Melosira* | |  | |  | |
| *Melosira varians* C. Agardh 1827 | | X | | X | |
| *Melosira* sp. | | X | |  | |
| **Subphylum** Bacillariophytina | |  | |  | |
| **Class** Mediophyceae | |  | |  | |
| **Subclass** Thalassiosirophycidae | |  | |  | |
| **Order** Thalassiosirales | |  | |  | |
| **Family** Thalassiosiraceae | |  | |  | |
| **Genus** *Stephanocyclus* | |  | |  | |
| *Stephanocyclus meneghinianus* (Kützing) Kulikovskiy, Genkal & Kociolek 2022 | | X | |  | |
| **Genus** *Thalassiosira* | |  | |  | |
| *Thalassiosira* sp. | | X | |  | |
| **Class** Bacillariophyceae | |  | |  | |
| **Subclass** Fragilariophycidae | |  | |  | |
| **Order** Fragilariales | |  | |  | |
| **Family** Fragilariaceae | |  | |  | |
| **Genus** *Fragilaria* | |  | |  | |
| *Fragilaria capucina* Desmazières 1830 | | X | |  | |
| *Fragilaria radians* (Kützing) D. M. Williams & Round 1988 | |  | | X | |
| *Fragilaria vaucheriae* (Kützing) J. B. Petersen 1938 | | X | | X | |
| *Fragilaria* sp. | | X | | X | |
| **Genus** *Fragilariforma* | |  | |  | |
| *Fragilariforma mesolepta* (Rabenhorst) Kharitonov 2005 | | X | | X | |
| *Fragilariforma virescens* (Ralfs) D. M. Williams & Round 1988 | | X | | X | |
| **Family** Staurosiraceae | |  | |  | |
| **Genus** *Pseudostaurosira* | |  | |  | |
| *Pseudostaurosira brevistriata* (Grunow) D. M. Williams & Round 1988 | | X | |  | |
| **Genus** *Stauroforma* | |  | |  | |
| *Stauroforma exiguiformis* (Lange-Bertalot) R. J. Flower, V. J. Jones & Round 1996 | | X | |  | |
| **Genus** *Staurosira* | |  | |  | |
| *Staurosira subsalina* (Hustedt) Lange-Bertalot 2004 | | X | |  | |
| **Order** Licmophorales | |  | |  | |
| **Family** Ulnariaceae | |  | |  | |
| **Genus** *Ctenophora* | |  | |  | |
| *Ctenophora pulchella* (Ralfs ex Kützing) D. M. Williams & Round 1986 | | X | | X | |
| **Genus** *Tabularia* | |  | |  | |
| *Tabularia fasciculata* (C. Agardh) D. M. Williams & Round 1986 | | X | | X | |
| **Genus** *Ulnaria* | |  | |  | |
| *Ulnaria acus* (Kützing) Aboal 2003 | | X | | X | |
| *Ulnaria ulna* (Nitzsch) Compère 2001 | | X | |  | |
| **Order** Rhabdonematales | |  | |  | |
| **Family** Tabellariaceae | |  | |  | |
| **Genus** *Asterionella* | |  | |  | |
| *Asterionella* sp. | | X | |  | |
| **Genus** *Diatoma* | |  | |  | |
| *Diatoma vulgaris* Bory 1824 | | X | | X | |
| **Genus** *Meridion* | |  | |  | |
| *Meridion constrictum* Ralfs 1843 | |  | | X | |
| **Subclass** Eunotiophycidae | |  | |  | |
| **Order** Eunotiales | |  | |  | |
| **Family** Eunotiaceae | |  | |  | |
| **Genus** *Actinella* | |  | |  | |
| *Actinella punctata* F.W. Lewis 1864 | |  | | X | |
| **Genus** *Eunotia* | |  | |  | |
| *Eunotia bilunaris* (Ehrenberg) Schaarschmidt 1880 | | X | | X | |
| *Eunotia faba* Ehrenberg 1837 | | X | |  | |
| *Eunotia incisa* W. Smith ex W. Gregory 1854 | | X | |  | |
| *Eunotia intermedia* (Krasske ex Hustedt) Nörpel & Lange-Bertalot 1993 | | X | |  | |
| *Eunotia mucophila* (Lange-Bertalot, Nörpel-Schempp & Alles) Lange-Bertalot 2007 | | X | |  | |
| *Eunotia* sp. | | X | | X | |
| **Subclass** Bacillariophycidae | |  | |  | |
| **Order** Achnanthales | |  | |  | |
| **Family** Achnanthaceae | |  | |  | |
| **Genus** *Achnanthes* | |  | |  | |
| *Achnanthes coarctata* (Brébisson ex W. Smith) Grunow 1880 | | X | |  | |
| **Family** Achnanthidiaceae | |  | |  | |
| **Genus** *Achnanthidium* | |  | |  | |
| *Achnanthidium minutissimum* (Kützing) Czarnecki 1994 | | X | | X | |
| *Achnanthidium petersenii* (Hustedt) C.E. Wetzel, Ector, D.M. Williams & Jüttner 2019 | |  | | X | |
| *Achnanthidium pyrenaicum* (Hustedt) H. Kobayasi 1997 | | X | |  | |
| *Achnanthidium* sp. | |  | | X | |
| **Genus** *Eucocconeis* | |  | |  | |
| *Eucocconeis* sp. | |  | | X | |
| **Genus** *Karayevia* | |  | |  | |
| *Karayevia clevei* (Grunow) Bukhtiyarova 1999 | |  | | X | |
| *Karayevia ploenensis* (Hustedt) Bukhtiyarova 1999 | | X | | X | |
| **Genus** *Planothidium* | |  | |  | |
| *Planothidium delicatulum* (Kützing) Round & Bukhtiyarova 1996 | | X | | X | |
| *Planothidium frequentissimum* (Lange-Bertalot) Lange-Bertalot 1999 | | X | | X | |
| *Planothidium lanceolatum* (Brébisson ex Kützing) Lange-Bertalot 1999 | | X | | X | |
| *Planothidium pumilum* Bąk & Lange-Bertalot 2015 | | X | | X | |
| **Genus** *Platessa* | |  | |  | |
| *Platessa conspicua* (Ant. Mayer) Lange-Bertalot 2004 | | X | | X | |
| *Platessa conspicua* (Ant. Mayer) Lange-Bertalot 2004 | | X | | X | |
| *Platessa oblongella* (Østrup) C.E. Wetzel, Lange-Bertalot & Ector 2017 | |  | | X | |
| *Platessa oblongella* (Østrup) C.E. Wetzel, Lange-Bertalot & Ector 2017 | | X | |  | |
| *Platessa saxonica* (Krasske ex Hustedt) Wetzel, Lange-Bertalot & Ector 2017 | | X | | X | |
| **Genus** *Psammothidium* | |  | |  | |
| *Psammothidium abundans* (Manguin) Bukhtiyarova & Round 1996 | | X | | X | |
| *Psammothidium daonense* (Lange-Bertalot) Lange-Bertalot 1999 | | X | |  | |
| *Psammothidium helveticum* (Hustedt) Bukhtiyarova & Round 1996 | |  | | X | |
| **Family** Cocconeidaceae | |  | |  | |
| **Genus** *Cocconeis* | |  | |  | |
| *Cocconeis euglypta* Ehrenberg 1854 | | X | | X | |
| *Cocconeis lineata* Ehrenberg 1843 | | X | | X | |
| *Cocconeis pediculus* Ehrenberg 1838 | | X | | X | |
| *Cocconeis placentula* Ehrenberg 1838 | | X | |  | |
| **Order** Naviculales | |  | |  | |
| **Family** Amphipleuraceae | |  | |  | |
| **Genus** *Amphipleura* | |  | |  | |
| *Amphipleura* sp. | | X | |  | |
| **Genus** *Frustulia* | |  | |  | |
| *Frustulia crassinervia* (Brébisson ex W. Smith) Lange-Bertalot & Krammer 1996 | | X | |  | |
| *Frustulia vulgaris* (Thwaites) De Toni 1891 | | X | | X | |
| **Family** Brachysiraceae | |  | |  | |
| **Genus** *Brachysira* | |  | |  | |
| *Brachysira microcephala* (Grunow) Compère 1986 | | X | |  | |
| **Family** Diadesmidaceae | |  | |  | |
| **Genus** *Luticola* | |  | |  | |
| *Luticola goeppertiana* (Bleisch) D. G. Mann ex J. Rarick, S. Wu, S. S. Lee & Edlund 2017 | | X | | X | |
| **Family** Diploneidaceae | |  | |  | |
| **Genus** *Diploneis* | |  | |  | |
| *Diploneis elliptica* (Kützing) Cleve 1894 | |  | | X | |
| *Diploneis* sp. | | X | | X | |
| **Family** Naviculaceae | |  | |  | |
| **Genus** *Caloneis* | |  | |  | |
| *Caloneis lancettula* (P. Schulz) Lange-Bertalot & Witkowski 1996 | | X | |  | |
| *Caloneis* sp. | |  | | X | |
| **Genus** *Genkalia* | |  | |  | |
| *Genkalia* sp. | |  | | X | |
| **Genus** *Gyrosigma* | |  | |  | |
| *Gyrosigma acuminatum* (Kützing) Rabenhorst 1853 | | X | | X | |
| *Gyrosigma attenuatum* (Kützing) Rabenhorst 1853 | |  | | X | |
| *Gyrosigma* sp. | |  | | X | |
| **Genus** *Navicula* | |  | |  | |
| *Navicula capitatoradiata* H. Germain ex Gasse 1986 | | X | | X | |
| *Navicula cryptotenella* Lange-Bertalot 1985 | | X | | X | |
| *Navicula gregaria* Donkin 1861 | | X | | X | |
| *Navicula lanceolata* Ehrenberg 1838 | | X | | X | |
| *Navicula metareichardtiana* Lange-Bertalot & Kusber 2019 | |  | | X | |
| *Navicula minuta* Skvortzov 1936 | | X | |  | |
| *Navicula rhynchotella* Lange-Bertalot 1993 | | X | |  | |
| *Navicula tripunctata* (O. F. Müller) Bory 1822 | | X | | X | |
| *Navicula* sp. | | X | |  | |
| **Family** Pinnulariaceae | |  | |  | |
| **Genus** *Pinnularia* | |  | |  | |
| *Pinnularia* cf. *abaujensis* (Pantocsek) R. Ross 1947 | | X | | X | |
| *Pinnularia apiculata* Gregory 1855 | |  | | X | |
| *Pinnularia gibba* (Ehrenberg) Ehrenberg 1843 | |  | | X | |
| *Pinnularia grunowii* Krammer 2000 | | X | |  | |
| **Family** Sellaphoraceae | |  | |  | |
| **Genus** *Fallacia* | |  | |  | |
| *Fallacia pygmaea* (Kützing) Stickle & D. G. Mann 1990 | | X | |  | |
| *Fallacia* *subhamulata* (Grunow) D. G. Mann 1990 | | X | | X | |
| **Genus** *Sellaphora* | |  | |  | |
| *Sellaphora bacillum* (Ehrenberg) D. G. Mann 2018 | |  | | X | |
| *Sellaphora capitata* D. G. Mann & S. M. McDonald 2004 | | X | | X | |
| *Sellaphora pupula* (Kützing) Mereschkovsky 1902 | | X | | X | |
| *Sellaphora saugerresii* (Desmazières) C. E. Wetzel & D. G. Mann 2015 | |  | | X | |
| *Sellaphora* sp. | | X | | X | |
| **Order** Cymbellales | |  | |  | |
| **Family** Cymbellaceae | |  | |  | |
| **Genus** *Brebissonia* | |  | |  | |
| *Brebissonia lanceolata* (C. Agardh) R. K. Mahoney & Reimer 1986 | |  | | X | |
| **Genus** *Cymbella* | |  | |  | |
| *Cymbella excisa* Kützing 1844 | | X | |  | |
| *Cymbella subcistula* Krammer 2002 | |  | | X | |
| *Cymbella subhelvetica* Krammer 2002 | | X | |  | |
| *Cymbella* sp. | | X | | X | |
| **Genus** *Encyonema* | |  | |  | |
| *Encyonema brevicapitatum* Krammer 1997 | | X | |  | |
| *Encyonema leibleinii* (C. Agardh) W. J.Silva, R. Jahn, T. A. V. Ludwig & M. Menezes 2013 | | X | | X | |
| *Encyonema minutum* (Hilse) D. G. Mann 1990 | | X | | X | |
| *Encyonema neogracile* Krammer 1997 | | X | | X | |
| *Encyonema reichardtii* (Krammer) D.G. Mann 1990 | | X | | X | |
| *Encyonema silesiacum* (Bleisch) D. G. Mann 1990 | | X | | X | |
| *Encyonema* sp. | |  | | X | |
| **Genus** *Encyonopsis* | |  | |  | |
| *Encyonopsis krammeri* E. Reichardt 1997 | | X | |  | |
| **Family** Cymbellales incertae sedis | |  | |  | |
| **Genus** *Gomphonella* | |  | |  | |
| *Gomphonella calcifuga* (Lange-Bertalot & E. Reichardt) A. Tuji 2020 | | X | |  | |
| *Gomphonella olivacea* (Hornemann) Rabenhorst 1853 | | X | | X | |
| **Family** Gomphonemataceae | |  | |  | |
| **Genus** *Gomphonema* | |  | |  | |
| *Gomphonema* cf. *apicatum* Ehrenberg 1854 | |  | | X | |
| *Gomphonema clavatulum* E. Reichardt 1999 | |  | | X | |
| *Gomphonema cuneolus* E. Reichardt 1997 | | X | |  | |
| *Gomphonema exilissimum* (Grunow) Lange-Bertalot & E. Reichardt 1996 | | X | | X | |
| *Gomphonema minutum* (C. Agardh) C. Agardh 1831 | |  | | X | |
| *Gomphonema parvulum* (Kützing) Kützing 1849 | | X | | X | |
| *Gomphonema pumilum* (Grunow) E. Reichardt & Lange-Bertalot 1991 | |  | | X | |
| *Gomphonema saprophilum* (Lange-Bertalot & E. Reichardt) Abarca, R. Jahn, J. Zimmermann & Enke 2014 | |  | | X | |
| *Gomphonema truncatum* Ehrenberg 1832 | | X | |  | |
| *Gomphonema varioreduncum* Jüttner, Ector, E. Reichardt, Van de Vijver & E.J. Cox 2013 | | X | | X | |
| *Gomphonema vibrio* Ehrenberg 1843 | |  | | X | |
| **Genus** *Reimeria* | |  | |  | |
| *Reimeria sinuata* (W. Gregory) Kociolek & Stoermer 1987 | | X | | X | |
| *Reimeria uniseriata* Sala, Guerrero & Ferrario 1993 | |  | | X | |
| **Family** Rhoicospheniaceae | |  | |  | |
| **Genus** *Rhoicosphenia* | |  | |  | |
| *Rhoicosphenia abbreviata* (C. Agardh) Lange-Bertalot 1980 | | X | | X | |
| *Rhoicosphenia* sp. | | X | | X | |
| **Order** Thalassiophysales | |  | |  | |
| **Family** Catenulaceae | |  | |  | |
| **Genus** *Amphora* | |  | |  | |
| *Amphora inariensis* Krammer 1980 | | X | | X | |
| *Amphora indistincta* Levkov 2009 | | X | |  | |
| *Amphora ovalis* (Kützing) Kützing 1844 | | X | |  | |
| *Amphora pediculus* (Kützing) Grunow 1875 | | X | | X | |
| **Genus** *Halamphora* | |  | |  | |
| *Halamphora veneta* (Kützing) Levkov 2009 | | X | |  | |
| **Order** Bacillariales | |  | |  | |
| **Family** Bacillariaceae | |  | |  | |
| **Genus** *Cymbellonitzschia* | |  | |  | |
| *Cymbellonitzschia diluviana* Hustedt 1950 | | X | |  | |
| **Genus** *Hantzschia* | |  | |  | |
| *Hantzschia amphioxys* (Ehrenberg) Grunow 1880 | | X | |  | |
| **Genus** *Nitzschia* | |  | |  | |
| *Nitzschia amphibia* Grunow 1862 | | X | | X | |
| *Nitzschia capitellata* Hustedt 1930 | | X | | X | |
| *Nitzschia dissipata* (Kützing) Rabenhorst 1860 | | X | | X | |
| *Nitzschia dubia* W. Smith 1853 | | X | | X | |
| *Nitzschia fonticola* (Grunow) Grunow 1881 | | X | | X | |
| *Nitzschia linearis* W. Smith 1853 | | X | | X | |
| *Nitzschia microcephala* Grunow 1880 | |  | | X | |
| *Nitzschia minuta* Blesich 1860 | |  | | X | |
| *Nitzschia palea* (Kützing) W. Smith 1856 | | X | | X | |
| *Nitzschia recta* Hantzsch ex Rabenhorst 1862 | | X | | X | |
| *Nitzschia sigmoidea* (Nitzsch) W. Smith 1853 | | X | | X | |
| *Nitzschia sociabilis* Hustedt 1957 | | X | | X | |
| *Nitzschia soratensis* E. A. Morales & M. L. Vis 2007 | | X | | X | |
| *Nitzschia* sp. | |  | | X | |
| **Genus** *Psammodictyon* | |  | |  | |
| *Psammodictyon panduriforme* (W. Gregory) D.G. Mann 1990 | | X | |  | |
| **Genus** *Tryblionella* | |  | |  | |
| *Tryblionella apiculata* W. Gregory 1857 | | X | | X | |
| **Order** Surirellales | |  | |  | |
| **Family** Rhopalodiaceae | |  | |  | |
| **Genus** *Epithemia* | |  | |  | |
| *Epithemia sorex* Kützing 1844 | | X | |  | |
| **Family** Surirellaceae | |  | |  | |
| **Genus** *Cymatopleura* | |  | |  | |
| *Cymatopleura elliptica* W. Smith 1851 | | X | | X | |
| **Genus** *Iconella* | |  | |  | |
| *Iconella roba* (Leclercq) Denys 2024 | |  | | X | |
| **Genus** *Surirella* | |  | |  | |
| *Surirella amphioxys* W. Smith 1856 | | X | |  | |
| *Surirella angusta* Kützing 1844 | | X | | X | |
| *Surirella brebissonii* Krammer & Lange-Bertalot 1987 | | X | | X | |
| *Surirella brebissonii* var. *kuetzingii* Krammer & Lange-Bertalot 1987 | | X | |  | |
| *Surirella lacrimula* J. D. English 2012 | | X | |  | |
| *Surirella minuta* Brébisson ex Kützing 1849 | |  | | X | |
| *Surirella undulata* (Ehrenberg) Ehrenberg 1845 | |  | | X | |

Table S5. Pairwise PERMANOVA results comparing seasonal communities (summer, autumn, winter) from urban pond sites.

| Group | R2 | *F* value | df | *p* value |
| --- | --- | --- | --- | --- |
| Summer v Autumn | 0.027 | 0.876 | 1, 32 | 0.632 |
| Summer v Winter | 0.075 | 2.667 | 1, 33 | 0 |
| Autumn v Winter | 0.072 | 2.416 | 1, 31 | 0.001 |

**Table S6.** Phytoplankton taxa were recorded for a single season (Summer, Autumn, or Winter) and across all seasons in the pond study sites.

| Taxonomical classification | Sum. | Aut. | Win. | Multiple seasons |
| --- | --- | --- | --- | --- |
| **Phylum** Cyanobacteria |  |  |  |  |
| **Class** Cyanophyceae |  |  |  |  |
| **Subclass** Nostocophycidae |  |  |  |  |
| **Order** Nostoclaes |  |  |  |  |
| **Family** Aphanizomenonaceae |  |  |  |  |
| **Genus** Aphanizomenon Morren ex Bornet & Flahault 1886 |  |  |  | X |
| **Genus** *Dolichospermum* (Bornet & Flahault) P.Wacklin, L.Hoffmann & Komárek 2009 |  |  |  | X |
| **Subclass** Oscillatoriophycidae |  |  |  |  |
| **Order** Oscillatoriales |  |  |  |  |
| **Family** Oscillatoriaceae |  |  |  |  |
| **Genus** Oscillatoria Vaucher ex Gomont 1892 | X |  |  |  |
| **Family** Microcoleaceae |  |  |  |  |
| **Genus** Planktothrix Anagnostidis & Komárek 1988 |  |  |  | X |
| **Order** Chroococcales |  |  |  |  |
| **Family** Microcystaceae |  |  |  |  |
| **Genus** *Aphanocapsa Nägeli 1849* |  |  |  | X |
| **Genus** *Aphanothece Nägeli 1849* |  |  |  | X |
| **Genus** *Microcystis Lemmermann 1907* |  |  |  | X |
| **Genus** *Snowella* Elenkin 1938 |  |  |  | X |
| **Genus** *Synechocystis Sauvageau 1892* |  |  |  | X |
| **Family** Chroococcaceae |  |  |  |  |
| **Genus** *Chroococcus* Nägeli 1849 |  |  |  | X |
| **Family** Microcystaceae |  |  |  |  |
| **Genus** *Merismopedia Meyen 1839* |  |  |  | X |
| **Order** Chroococcidiopsidales |  |  |  |  |
| **Family** Gloeocapsaceae |  |  |  |  |
| **Genus** *Gloeocapsa Kützing 1843* |  |  |  | X |
| **Phylum** Charophyta |  |  |  |  |
| **Class** Klebsormidiophyceae |  |  |  |  |
| **Order** Klebsormidiales |  |  |  |  |
| **Family** Elakatotrichaceae |  |  |  |  |
| **Genus** Elakatothrix Wille 1898 |  | X |  |  |
| **Class** Zygnematophyceae |  |  |  |  |
| **Subclass** Zygnematophycidae |  |  |  |  |
| **Order** Spirogyrales |  |  |  |  |
| **Family** Spirogyraceae |  |  |  |  |
| **Genus** *Spirogyra* Link 1820 | X |  |  |  |
| **Order** Desmidiales |  |  |  |  |
| **Family** Closteriaceae |  |  |  |  |
| **Genus** Closterium Nitzsch ex Ralfs 1848 |  |  |  | X |
| **Family** Desmidiaceae |  |  |  |  |
| **Genus** Cosmarium Corda ex Ralfs 1848 |  |  |  | X |
| **Genus** Staurastrum Meyen ex Ralfs 1848 |  |  |  | X |
| **Phylum** Cryptophyta |  |  |  |  |
| **Class** Cryptophyceae |  |  |  |  |
| **Order** Pyrenomonadales |  |  |  |  |
| **Family** Pyrenomonadaceae |  |  |  |  |
| **Genus** *Rhodomonas* G.Karsten 1898 |  |  | X |  |
| **Order** Cryptomonadales |  |  |  |  |
| **Family** Cryptomonadaceae |  |  |  |  |
| **Genus** *Cryptomonas* Ehrenberg 1831 |  |  |  | X |
| **Phylum** Ochrophyta |  |  |  |  |
| **Class** Chrysophyceae |  |  |  |  |
| **Order** Chromulinales |  |  |  |  |
| **Family** Dinobryaceae |  |  |  |  |
| **Genus** *Dinobryon* Ehrenberg 1834 |  |  |  | X |
| **Phylum** Myzozoa |  |  |  |  |
| **Class** Dinophyceae |  |  |  |  |
| **Subclass** Peridiniphycidae |  |  |  |  |
| **Order** Gonyaulacales |  |  |  |  |
| **Family** Ceratiaceae |  |  |  |  |
| **Genus** *Ceratium* F.Schrank 1793 |  |  |  | X |
| **Order** Gymnodiniales |  |  |  |  |
| **Family** Gymnodiniaceae |  |  |  |  |
| **Genus** *Gymnodinium* F.Stein 1878 |  |  |  | X |
| **Order** Peridiniales |  |  |  |  |
| **Family** Peridiniaceae |  |  |  |  |
| **Genus** *Peridinium* Ehrenberg 1830 |  |  |  | X |
| **Phylum** Euglenozoa |  |  |  |  |
| **Class** Euglenophyceae |  |  |  |  |
| **Subclass** Euglenophycidae |  |  |  |  |
| **Order** Euglenales |  |  |  |  |
| **Family** Euglenaceae |  |  |  |  |
| **Genus** *Euglena* Ehrenberg 1830 |  |  |  | X |
| **Genus** *Trachelomonas* Ehrenberg 1834 |  |  |  | X |
| **Family** Phacaceae |  |  |  |  |
| **Genus** *Phacus* Dujardin 1841 |  |  |  | X |
| **Phylum** Bacillariophyta |  |  |  |  |
| **Class** Bacillariophyceae |  |  |  |  |
| **Subclass** Bacillariophycidae |  |  |  |  |
| **Order** Achnanthales |  |  |  |  |
| **Family** Cocconeidaceae |  |  |  |  |
| **Genus** *Cocconeis* Ehrenberg 1837 |  |  |  | X |
| **Order** Bacillariales |  |  |  |  |
| **Family** Bacillariaceae |  |  |  |  |
| **Genus** *Nitzschia* Hassall 1845 |  |  |  | X |
| **Order** Cymbellales |  |  |  |  |
| **Family** Cymbellaceae |  |  |  |  |
| **Genus** *Cymbella* C.Agardh 1830 | X |  |  |  |
| **Genus** *Placoneis* Mereschkowsky 1903 | X |  |  |  |
| **Family** Gomphonemataceae |  |  |  |  |
| **Genus** *Gomphonema* Ehrenberg 1832 |  |  |  | X |
| **Family** Rhoicospheniaceae |  |  |  |  |
| **Genus** *Rhoicosphenia* Grunow 1860 | X |  |  |  |
| **Order** Naviculales |  |  |  |  |
| **Family** Amphipleuraceae |  |  |  |  |
| **Genus** *Frustulia* Rabenhorst 1853 |  | X |  |  |
| **Family** Naviculaceae |  |  |  |  |
| **Genus** *Gyrosigma* Hassall 1845 |  | X |  |  |
| **Genus** *Navicula* Bory 1822 |  |  |  | X |
| **Family** Pinnulariaceae |  |  |  |  |
| **Genus** *Pinnularia* Ehrenberg 1843 |  |  |  | X |
| **Order** Surirellales |  |  |  |  |
| **Family** Surirellaceae |  |  |  |  |
| **Genus** *Cymatopleura* W.Smith 1851 |  |  |  | X |
| **Genus** *Surirella* Turpin 1828 |  | X |  |  |
| **Order** Thalassiophysales |  |  |  |  |
| **Family** Catenulaceae |  |  |  |  |
| **Genus** Amphora Ehrenberg ex Kützing 1844 |  |  |  | X |
| **Subclass** Fragilariophycidae |  |  |  |  |
| **Order** Fragilariales |  |  |  |  |
| **Family** Fragilariaceae |  |  |  |  |
| **Genus** *Fragilaria* Lyngbye 1819 |  |  |  | X |
| **Genus** *Synedra* Ehrenberg 1830 | X |  |  |  |
| **Order** Licmophorales |  |  |  |  |
| **Family** Ulnariaceae |  |  |  |  |
| **Genus** *Ulnaria* (Kützing) Compère 2001 |  |  |  | X |
| **Order** Rhabdonematales |  |  |  |  |
| **Family** Tabellariaceae |  |  |  |  |
| **Genus** *Asterionella* Hassall 1850 |  |  |  | X |
| **Genus** *Diatoma* Bory 1824 | X |  |  |  |
| **Genus** Tabellaria Ehrenberg eX Kützing 1844 |  |  |  | X |
| **Class** Coscinodiscophyceae |  |  |  |  |
| **Order** Aulacoseirales |  |  |  |  |
| **Family** Aulacoseiraceae |  |  |  |  |
| **Genus** *Aulacoseira* Thwaites 1848 |  |  |  | X |
| **Subclass** Melosirophycidae |  |  |  |  |
| **Order** Melosirales |  |  |  |  |
| **Family** Melosiraceae |  |  |  |  |
| **Genus** *Melosira* C.Agardh 1824 |  |  |  | X |
| **Class** Mediophyceae |  |  |  |  |
| **Subclass** Thalassiosirophycidae |  |  |  |  |
| **Order** Stephanodiscales |  |  |  |  |
| **Family** Stephanodiscaceae |  |  |  |  |
| **Genus** *Cyclotella* (Kützing) Brébisson 1838 |  |  |  | X |
| **Genus** *Stephanodiscus* Ehrenberg 1845 |  |  |  | X |
| **Phylum** Chlorophyta |  |  |  |  |
| **Class** Chlorophyceae |  |  |  |  |
| **Order** Chlamydomonadales |  |  |  |  |
| **Family** Chlamydomonadaceae |  |  |  |  |
| **Genus** *Chlamydomonas* Ehrenberg 1833 |  |  |  | X |
| **Family** Tetrasporaceae Wittrock 1872 |  |  |  |  |
| **Genus** Tetraspora Link ex Desvaux |  |  |  | X |
| **Family** Goniaceae | X |  |  |  |
| **Genus** *Gonium* O.F. Müller 1773 |  |  |  |  |
| **Family** Sphaerocystidaceae |  |  |  | X |
| **Genus** *Sphaerocystis* R.Chodat 1897 |  |  |  |  |
| **Family** Volvocaceae | X |  |  |  |
| **Genus** *Eudorina* Ehrenberg 1832 | X |  |  |  |
| **Genus** *Pandorina* Bory 1826 | X |  |  |  |
| **Genus** Volvox Linnaeus 1758 |  |  |  |  |
| **Order** Sphaeropleales |  |  |  |  |
| **Family** Hydrodictyaceae |  |  |  | X |
| **Genus** *Pediastrum* Meyen 1829 |  |  |  | X |
| **Genus** *Tetraedron* Kützing 1845 |  |  |  |  |
| **Family** Neochloridaceae |  |  |  | X |
| **Genus** *Golenkinia* Chodat 1894 |  |  |  |  |
| **Family** Radiococcaceae |  | X |  |  |
| **Genus** *Gloeocystis* Nägeli 1849 |  |  |  |  |
| **Family** Scenedesmaceae |  |  |  | X |
| **Genus** *Coelastrum* Nägeli 1849 |  |  |  | X |
| **Genus** *Crucigenia* Morren 1830 |  |  |  | X |
| **Genus** *Scenedesmus* Meyen 1829 |  |  |  | X |
| **Genus** *Tetrastrum* Chodat 1895 |  |  |  |  |
| **Family** Selenastraceae |  |  |  | X |
| **Genus** *Ankistrodesmus* Corda 1838 |  |  |  | X |
| **Genus** *Kirchneriella* Schmidle 1893 |  |  |  | X |
| **Genus** *Monoraphidium* Komárková-Legnerová 1969 |  |  |  | X |
| **Genus** *Selenastrum* Reinsch 1866 |  |  |  |  |
| **Class** Trebouxiophyceae |  |  |  |  |
| **Order** Chlorellales |  |  |  |  |
| **Family** Chlorellaceae | X |  |  |  |
| **Genus** *Actinastrum* Lagerheim 1882 |  |  |  | X |
| **Genus** *Chlorella* Beyerinck [Beijerinck] 1890 |  | X |  |  |
| **Genus** *Closteriopsis* Lemmermann 1899 |  |  |  | X |
| **Genus** *Dictyosphaerium* Nägeli 1849 |  |  |  | X |
| **Genus** *Micractinium* Fresenius 1858 |  |  |  |  |
| **Family** Oocystaceae |  | X |  |  |
| **Genus** *Crucigeniella* Lemmermann 1900 |  | X |  |  |
| **Genus** *Lagerheimia* R.Chodat 1895 |  |  |  | X |
| **Genus** Oocystis Nägeli eX A.Braun 1855 |  |  |  |  |

**Table S6**. Water quality thresholds in lowland water bodies (Ryding and Raast, 1989; UKTAG 2022).

| **Category**  **(Lakes)** | **Nitrate NO3-N mg L¯¹** | **Ammonium NH4-N mg L¯¹** | **Total Phosphorus µg L¯¹** | **Trophic category** |
| --- | --- | --- | --- | --- |
| Low | <1.0 | - | 5-10 | Oligotrophic |
| Medium | 1.0-2.0 | 0.2-0.6 | 10-35 | Mesotrophic |
| High | 2.0-3.5 | 0.6-1 | 35-100 | Eutrophic |
| Very high | >3.5 | >1 | >100 | Hypereutrophic |
| **WFD standards of surface water quality status** | **pH** | **Ammonium NH4-N mg L¯¹** | **Orthophosphate µg L¯¹** | **Dissolved oxygen %** |
| High | ≥6 to ≤9 | 0.3 | 19 | 70 |
| Good |  | 0.6 | 40 | 60 |
| Moderate |  | 1.1 | 114 | 54 |
| Poor |  | 2.5 | 842 | 45 |
| **Category**  **(inland waters)** | **Zinc µg L¯¹** | **Mg mg L¯¹** | **Alkalinity mg L¯¹ CaCO3** |  |
| Low | - | 0-75 | 20-50 |  |
| Medium | - | 75-150 | 50-150 |  |
| High | 7.8-10.9 | 150-300 | >150 |  |
|  | - | >300 | - |  |
